# River-specific macrogenomic diversity in *Simulium guianense s*. *l*. (Diptera: Simuliidae), a complex of tropical American vectors associated with human onchocerciasis

**DOI:** 10.1371/journal.pone.0181679

**Published:** 2017-07-20

**Authors:** Peter H. Adler, Neusa Hamada, Jeane Marcelle Cavalcante do Nascimento, Maria Eugenia Grillet

**Affiliations:** 1 Department of Plant and Environmental Sciences, Clemson University, Clemson, South Carolina, United States of America; 2 Instituto Nacional de Pesquisas da Amazônia, Coordenação de Biodiversidade, Curso de pós-graduação em Entomologia, Manaus, Amazonas, Brazil; 3 Laboratorio de Biología de Vectores y Parásitos, Instituto de Zoología y Ecología Tropical, Facultad de Ciencias, Universidad Central de Venezuela, Caracas, Venezuela; Universita degli Studi di Roma La Sapienza, ITALY

## Abstract

*Simulium guianense* Wise is a Latin American vector complex of black flies associated with transmission of the causal agent of human onchocerciasis (river blindness). An analysis of the chromosomal banding patterns of 607 larvae of *S*. *guianense s*. *l*. revealed a high level of variation involving 83 macrogenomic rearrangements across 25 populations in Brazil, French Guiana, and Venezuela. The 25 populations were assigned to 13 cytoforms (A1, A2, B1–B4, C, D, E1–E4, and F), some of which are probably valid species. Based on geographical proximity, a member of the B group of cytoforms probably represents the name-bearing type specimen of *S*. *guianense* and the primary vector in the last-remaining onchocerciasis foci in the Western Hemisphere. Cytoform B3 in Amapá State is implicated as an anthropophilic simuliid in an area currently and historically free of onchocerciasis. Distributions of cytoforms are associated with geography, elevation, and drainage basin, and are largely congruent with ecoregions. Despite extraordinarily large larval populations of *S*. *guianense s*. *l*. in big rivers and consequent production of female flies for dispersal, the cytoforms maintain their chromosomal distinction within individual rivers, suggesting a high degree of fidelity to the specialized breeding habitats—rocky shoals—of the natal rivers.

## Introduction

Cryptic species and genetically differentiated forms are common in medically important Diptera [1–4]. The largest aggregates of cryptic species among blood-feeding arthropods are in the family Simuliidae, and include species complexes responsible for vector-borne diseases, such as human onchocerciasis or river blindness [5]. The discovery that simuliid vectors are complexes of multiple, structurally cryptic species ranks among the significant advancements in the battle against onchocerciasis [6]. Cryptic taxa typically differ in breeding habitats, geographical distributions, vectorial capacity, and other traits of significance for ecology, epidemiology, and vector control [1,7,8].

Human onchocerciasis was introduced into the New World via the slave trade, eventually becoming established in six countries [9]. In the Americas, this parasitic disease was formerly prevalent in 13 endemic foci in Brazil, Colombia, Ecuador, Guatemala, Mexico, and Venezuela, where more than half a million people were considered at risk of infection [10]. By 2014, a relentless mass-distribution program of ivermectin had interrupted or eliminated transmission in 11 of the 13 Latin American foci [11]. Challenges remain in the two Amazonian foci straddling Brazil and Venezuela, where more than 29,500 people, mostly of the semi-nomadic indigenous Yanomami population, live in an active transmission zone [10,12]. Transmission of the disease agent, *Onchocerca volvulus*, occurs in the lowlands (0–500 m above sea level, asl) and uplands (500–1200 m asl) of the Amazonian region in Brazil and Venezuela [12,13], where *Simulium guianense* Wise is the predominant human-biting black fly and main vector of *O*. *volvulus* in the hyperendemic and mesoendemic areas [12,14,15]. The immature stages of *S*. *guianense* inhabit primarily large, swift, open-canopy rivers [13,15].

Geographical variation in the epidemiological importance and biting behavior of *S*. *guianense*, such as the degree of anthropophily versus zoophily [14,16], provided early hints of a species complex. A chromosomal analysis of *S*. *guianense* provided additional evidence of hidden diversity, revealing four cytological forms, based on five collections [17]. The nearly one-to-one correspondence between the number of cytologically distinct populations and the number of collections suggested additional forms across the distribution [18] of *S*. *guianense* in Brazil and other northern South American countries. A subsequent chromosomal study of three more populations, coupled with a molecular barcoding analysis of eight populations, provided further evidence of diversity within *S*. *guianense* [19], as did additional barcoding studies [20,21].

To determine the extent and patterns of differentiation within the *S*. *guianense* complex, we conducted a geographically broad macrogenomic study, based on rearrangements of the polytene chromosomes in the larval silk glands. Our study complements that of Crainey et al. [22], who used a molecular approach to examine genetic structure of *S*. *guianense* at eight sites, most of which correspond to sites in our study area.

## Materials and methods

### Ethics statement

In Brazil, black fly collections were made on public or owner-permitted private lands. A permit to collect zoological specimens was provided by SISBIO (permanent permit number 10873–1 to NH). In French Guiana, collecting was arranged by Institut Pasteur. In Venezuela, a special collection permit from INPARQUES (National Institute of Natural Parks) allowed us to sample black flies in the Gran Sabana region of southern Venezuela. No collections involved endangered or protected species.

### Study area

**Fig 1:**
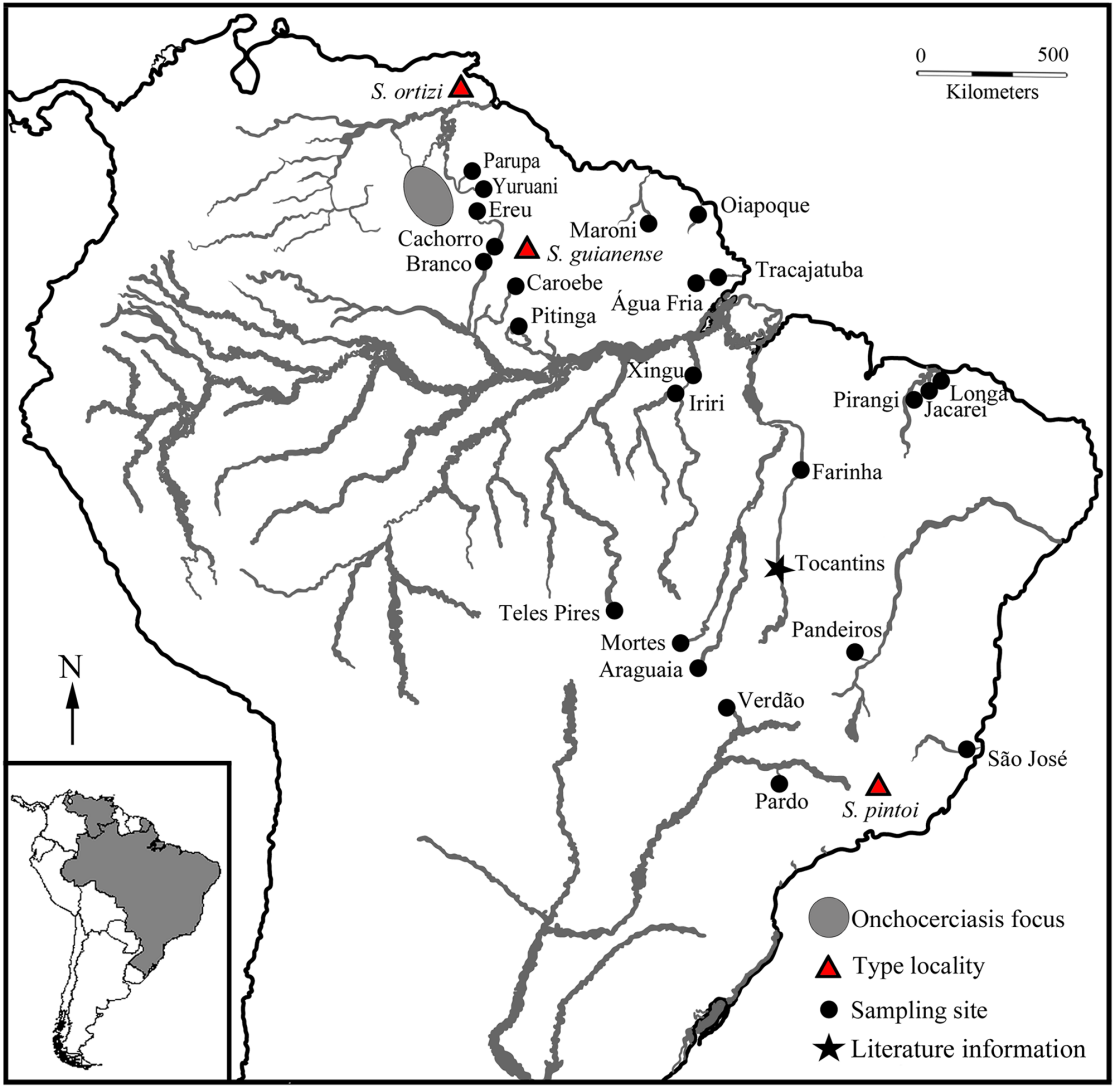
Major rivers of Brazil, French Guiana, and Venezuela, showing sampling sites for *Simulium guianense s*. *l*. Sampling sites are named by river, corresponding to names used in the text and Table 1. Type localities of *S*. *guianense*, *S*. *ortizi*, and *S*. *pintoi*, and current onchocerciasis foci are indicated.

**Fig 2:**
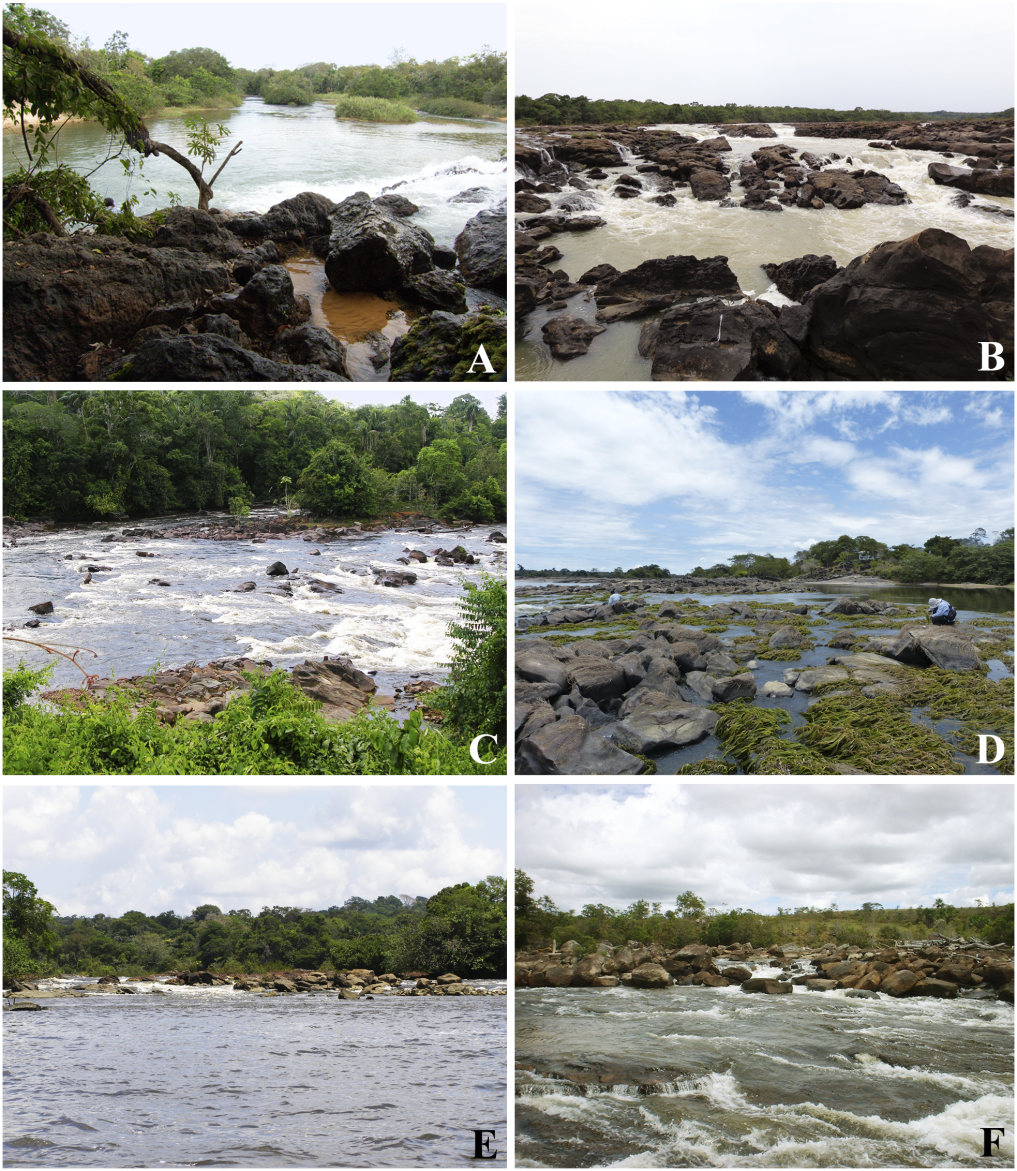
Sampling sites for *Simulium guianense* cytoforms A and B. A. Pandeiros River, Minas Gerais state. B. Teles Pires River, Mato Grosso state. C. Pitinga River, Amazonas state. D. Branco River, Roraima state. E. Oiapoque River, Amapá state. F. Yuruani River, Bolivar state.

**Fig 3:**
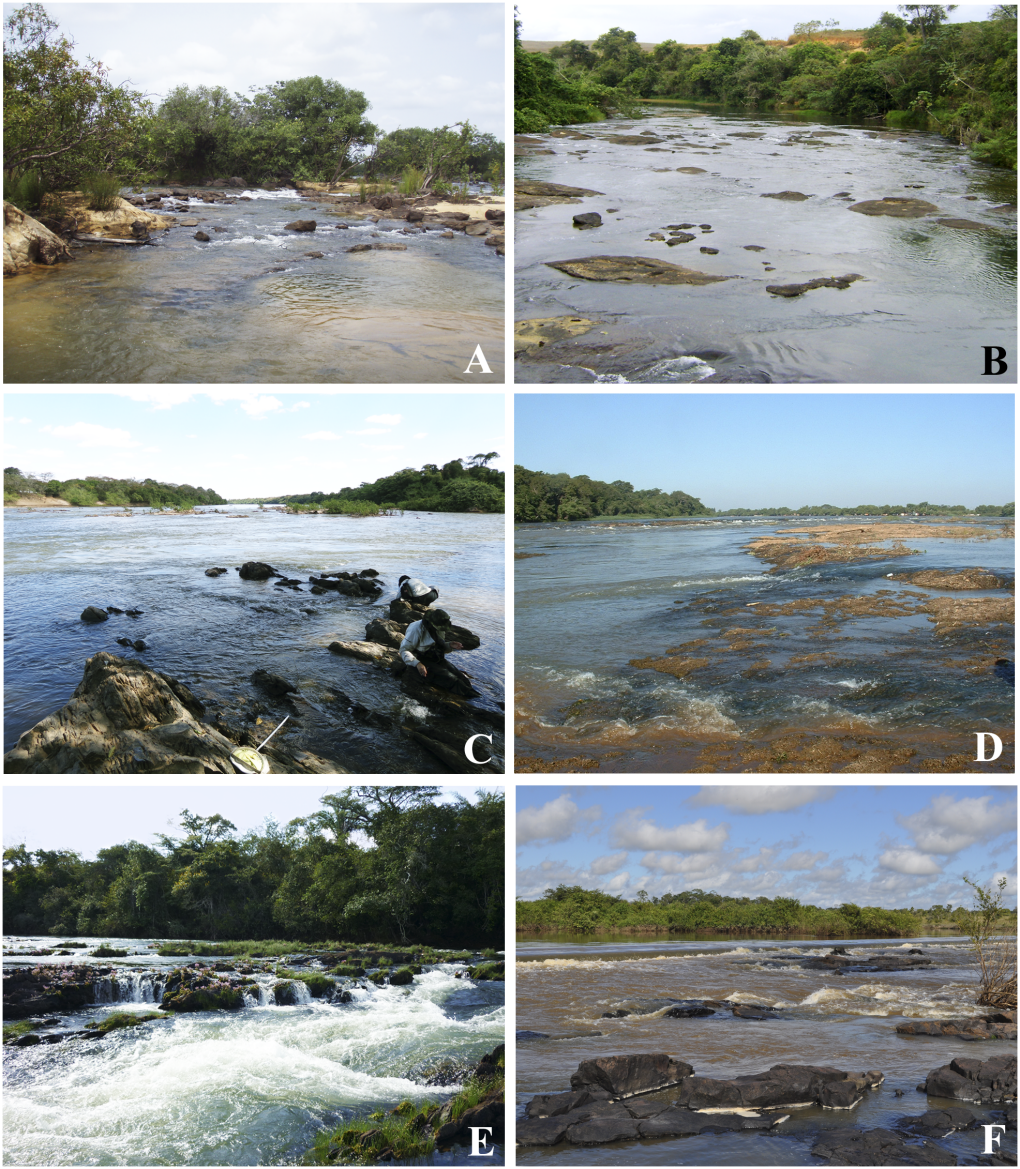
Sampling sites for *Simulium guianense* cytoforms D, E, and F. A. Xingu River, Pará state. B. São José River, Espírito Santo state. C. Araguaia River, Mato Grosso state. D. Pardo River, São Paulo state. E. Mortes River, Mato Grosso state. F. Longá River, Piauí state.

In Brazil, *S*. *guianense s*. *l*. (*sensu lato*) was collected in 12 states representing four geographic regions: four in the North Region, three in the Northeast, two in the Central-West, and three in the Southeast (Table 1; Figs 1–3). Although *S*. *guianense s*. *l*. has been reported in Paraná and Santa Catarina states in the South region [18], we did not find it despite our collecting efforts in the three states of this region. Sites with *S*. *guianense s*. *l*. were located in the Amazônia, Atlantic Forest (Mata Atlântica), Caatinga, and Cerrado biomes (Table 1). In French Guiana, *S*. *guianense* was collected from the Maroni River, which originates on the northern slope of the Tumucumaque Mountains near the Brazilian border and runs through a dense tropical rain forest to the Atlantic Ocean [23]. In Venezuela, *S*. *guianense s*. *l*. was collected in Canaima National Park of the Gran Sabana region in the southeastern corner of Bolívar state [24]. Most of the Gran Sabana uplands lie between 500 and 1500 m above sea level. The area is covered mainly by treeless savannas interspersed with montane and gallery forests.

**Table 1:** Collection sites for larvae of *Simulium guianense s*. *l*. analyzed chromosomally.

Country, Region, State	Drainage Basin	Location	River Width (m)	Water Temp. (°C)	Elevation (m)	Collection Date	Cytoform (*n*)^1^
**Brazil**							
**North**							
Amapá	Oiapoque	Oiapoque, Rio Oiapoque, Cachoeira Grande, 03°48'13"N 51°52'31"W	700	27.8	7	9/VIII/2011	B3 (63)
Amapá	Araguari	Serra do Navio, Rio Água Fria, 00°48'34"N 51°58'45"W	20	24.8	78	3/VIII/2011	B2 (8)
Amapá	Araguari	Amapá, Ferreira Gomes, Rio Tracajatuba, 01°01'22"N 51°05'35"W	10	26.3	14	11/VIII/2011	B3 (18)
Amazonas	Amazonas	Presidente Figueiredo, below dam in Rio Pitinga, Cachoeira 40 ilhas, 00°53'39"S 59°34'45"W	50	29.0	66	7/XII/2006	B2 (31)
Pará	Xingu	Altamira, Rio Xingu, Quebra Canela, downstream of Altamira, 03°18'06"S 52°02'34"W	4900	30.4	86	26/X/2007	D (42)
Pará	Xingu	Altamira, Rio Iriri, 03°49'16"S 52°38'16"W	1000	31.5	122	25/X/2007	D (9)
Roraima	Amazonas	Caroebe, Ramal 37, Rio Caroebe, Fazenda Cachoeirinha, 00°57'09"N 59°37'00"W	20	28.2	146	23/III/2012	B2 (25)
Roraima	Branco	Amajari, Fazenda São Sebastião, Rio Ereu, 04°02'00"N 61°23'10"W	30	31.3	143	26/III/2012	B2 (24)
Roraima	Branco	Caracaraí, Rio Branco, Cachoeira Bem Querer, 01°55'07"N 61°00'01"W	600	29.0	48	20/III/2013	B2 (7)
Roraima	Branco	Cantá, Rio Cachorro, bridge, 02°25'19"N 60°40'02"W	20	28.0	66	28/III/2012	B2 (27)
**Northeast**							
Ceará	Parnaíba	Rio Pirangi, waterfall Pirapora, 03°33'32"S 41°21'57"W	20	26.1	355	6/II/2011	F (10)
Maranhão	Tocantins	Carolina, Rio Farinha, 06°51'49"S 47°28'08"W	450	25.0	168	16/XI/2000, 11/VIII/2001	C (32)
Piauí	Parnaíba	Barras, Rio Longá, near bridge, 04°12'16"S 42°14'21"W	170	29.6	75	27, 28/V/2011	F (29)
Piauí	Parnaíba	Piracuruca, Rio Jacareí, BR 343, bridge, 03°43'59"S 41°40'54"W	20	28.4	74	2/VI/2011	F (11)
**Central-West**							
Góias^2^	Tocantins	Rio Tocantins and Rio Mucambão, near Minaçu	-	-	305	11, 14/XI/1991	A1 (36)
Góias	Paraná	Rio Verde, Rio Verdão, 17°32'34"S 50°33'33"W	150	20.0	523	20/V/2006	E4 (43)
Mato Grosso	Araguaia	Nova Xavantina, Travessão das facas, Rio das Mortes, 14°48'03"S 52°38'38"W	200	22.9	276	7/VII/2012	E3 (15)
Mato Grosso	Araguaia	Barra do Garça, Rio Araguaia, Travessão da Pitomba, 15°53'07"S 52°06'10"W	210	24.0	293	13/VII/2012	E2 (45)
Mato Grosso	Tapajós	Santa Rita do Trivelato, Rio Teles Pires, Salto Magessi, 13°34'34"S 55°16'00"W	160	29.2	356	2/XI/2012	B1 (23)
**Southeast**							
Espírito Santo	Doce	Sooretama, Rio São José, 19°07'33''S 40°14'26''W	60	-	36	3/V/2013	E1 (30)
Minas Gerais	São Francisco	Januaria, Rio Pandeiros (cachoeira), 15°30'42"S 44°45'11"W	32	23.5	500	2/VI/2014	A2 (21)
São Paulo	Paraná	Terra Roxa, Rio Pardo, 20°48'20"S 48°15'22"W	200	26.0	477	17/IX/2005	E3 (20)
**French Guiana**	Maroni	Maripasoula, Rio Maroni, 03°27'53"N 54°00'15"W	130	26.0	106	17/VI/1999	B2 (22)
**Venezuela**							
Bolívar	Caroni	Grand Savana National Park, Rio Parupa, 05°40'38"N 61°32'43"W	10	22.9	1270	15/III/2007	B4 (38)
Bolívar	Caroni	Grand Savana National Park, Rio Yuruani, 05°05'16"N 61°05'56"W	30	24.5	870	12/III/2007	B4 (14)

^1^ *n* = Number of larvae completely analyzed chromosomally.

^2^ The two Minaçu (Góias state) sites of Charalambous et al. [17] were not sampled in the present study, but are included as a single entry for completeness; river width and temperature were not stated.

To provide context for interpreting our results, we use the freshwater ecoregions based on the composition and distribution of freshwater fish species [25]. The ecoregions in which our collections are represented are briefly characterized as follows [26]:

#### Amazonas Estuary & Coastal Drainages

This ecoregion, in the Amazônia biome, lies mostly below 100 m asl and includes large river deltas in the lower Amazon River basin and adjacent coastal drainages that enter the Atlantic Ocean.

#### Amazonas Guiana Shield

Located in the Amazônia biome, this ecoregion includes the river basins that drain much of the southern slopes of the Guiana Shield and flow into the Amazon River. The rivers are black, clear, or whitewater, with elevations reaching 2100 m asl.

#### Guianas

Included in the Amazônia biome, this ecoregion comprises the predominantly flat coastal lowlands of the Guiana Shield, reaching elevations of 1286 m asl. It includes rivers that flow into the Atlantic Ocean from the northern and eastern slopes of the Guiana Shield.

#### Northeastern Mata Atlantica

Located in the Atlantic Forest biome, this ecoregion comprises all coastal drainages in eastern Brazil, with the rivers flowing into the Atlantic Ocean. The area has a diverse landscape, such as plateaus, valleys, and crystalline mountains reaching elevations of 2890 m asl.

#### Orinoco Guiana Shield

Located in the Amazônia biome, this area includes the lowlands and highlands of the Guiana Shield, with forested lowlands, rolling high plains, rounded hills, and tepuis. The streams and rivers are typically clear or blackwater and often have rapids and waterfalls; they flow into the Río Orinoco and Atlantic Ocean. Elevations reach 2800 m asl.

#### Parnaiba

Located in the Caatinga and Cerrado biomes, this ecoregion covers the drainage basin of the Rio Parnaíba and coastal drainages, with elevations ranging from sea level to more than 900 m asl. Its rivers flow into the Atlantic Ocean.

#### São Francisco

Occupying areas in three biomes—Cerrado, Caatinga, and Atlantic Forest (the smallest portion)—this ecoregion encompasses all drainages of the São Francisco River basin [27], from sea level to 1000 m asl.

#### Tapajós—Juruena

This ecoregion includes areas in the Amazônia biome in the north and the Cerrado in the south. The basin of the Rio Tapajós and its tributaries constitute the freshwater environment of this area, with drainages flowing into the Amazon River. Elevations range from 28 m to 873 m asl.

#### Tocantins—Araguaia

This ecoregion includes the Tocantins and Araguaia river basins in the Amazônia and Cerrado biomes. The drainages flow into the Rio Pará, Amazon estuary, or Atlantic Ocean. The heterogeneous landscape ranges from 25 m to 1600 m asl.

#### Upper Paraná

This ecoregion covers areas in the Atlantic Forest and Cerrado biomes from about 120 m to more than 1500 m asl. The basin of the upper Rio Paraná and its tributaries constitute the freshwater environment of this ecoregion. The drainages flow into the Lower Paraná River.

#### Xingu

Located in the Amazônia (about 90% of the total area) and Cerrado biomes, the Xingu ecoregion includes the basins of the Rio Xingu, with drainages flowing into the Amazon River. Elevations reach 800 m asl.

### Collection of larvae

Larvae of *S*. *guianense s*. *l*. were collected at 25 sites in Brazil, French Guiana, and Venezuela, representing 11 ecoregions and a range of elevations (7–1270 m asl), river widths (10–4900 m), and water temperatures (20.0°–31.5°C) (Table 1, Fig 1). Samples were taken with forceps from the leaves of the Podostemaceae or, where the plants were scarce, from rocks (Longá River), sticks (Yuruani River), or rocks and leaves (Pirangi River). The samples were fixed in 3 changes of 1:3 acetic ethanol and held at -4°C until processing.

### Chromosome preparation and interpretation

Larvae were split posteroventrally and Feulgen-stained following the procedures of Charalambous et al. [17]. Silk glands with stained polytene chromosomes, plus one gonad for gender determination [28], were placed in a drop of 50% acetic acid and flattened under a coverslip by thumb pressure. Representative larvae from each collection were deposited in the Invertebrate Collection of the Instituto Nacional de Pesquisas da Amazônia, Manaus, Brazil, and the Invertebrate Collection of Laboratorio de Biología de Vectores y Parásitos, Instituto de Zoología y Ecología Tropical, Caracas, Venezuela.

Photographs of high-quality chromosome preparations were taken under oil immersion on an Olympus BX40 or BX51 compound microscope. Photographic negatives were scanned with a Nikon Coolscan V and imported into Adobe^®^ PhotoShop^®^ Elements 8 to prepare chromosomal maps. Photographic negatives of chromosomes were deposited in the Clemson University Arthropod Collection, Clemson, SC.

As customary [29], we based our standard map on the predominant and most central sequence for the short (S) and long (L) arms of each chromosome (I, II, and III) in the *S*. *guianense* complex. Although we followed the conventions of Charalambous et al. [17] for constructing the standard map, the newly discovered diversity of band sequences in our samples required modification of the original standard map to represent the central and most predominant sequences. IIL-4, IIL-6, and IIIL-3 were shown as homozygous standard by Charalambous et al. [17] in all larvae of their two Toncantins (Minaçu) populations (cytoform A1) and in their corresponding figures (Figures 5 and 6 in [17]). However, only the Minaçu populations and one other similar population (Pandeiros River) had these sequence polarities among all studied populations. We, therefore, adopted an opposite interpretation and considered the Minaçu (and Rio Pandeiros) populations as inverted for *IIL-4*, *IIL-6*, and *IIIL-3*, rendering all other populations standard for these sequences. The section numbers within these inversions were renumbered to provide consecutive sequences for the revised standard map.

Selected landmarks labeled on our maps follow the nomenclature of Rothfels et al. [29]. IIS, for instance, includes the trapezoidal, Ring of Balbiani, bulge, and shoestring markers. All rearrangements that were found in our samples are indicated on our chromosomal maps by brackets or arrows. Inversions fixed in a cytoform are italicized in the text and underlined on our maps. Numbering of inversions follows that used by Charalambous et al. [17]; newly discovered inversions continue the numbering sequence in each arm. Polymorphic (floating) rearrangements, including those linked to sex, are in Roman type. Polymorphisms with a minimum of four larvae in each of three band-sequence categories (ss, si, ii, where s = standard, i = inverted) were tested for linkage to gender; if sex linkage was not significant (P > 0.05), the polymorphisms were tested for Hardy-Weinberg equilibrium.

We use “cytoform” as a neutral term for a chromosomally cohesive and distinguishable entity based on one or more chromosomal features; no statement about reproductive isolation is implied. We designate cytoforms with letters, according to the precedent established by Charalambous et al. [17]. To encode additional information regarding relationships, we also designated some cytoforms with a number following a letter (e.g., B1, B2, B3, and B4); thus, for example, each numbered member of the B group is chromosomally distinct but shares unique chromosomal characters with other B group members.

### Relationships among cytoforms

A cladistic approach was used to infer relationships among cytoforms. We used shared chromosomal inversions, relative to the standard sequence, as evidence of common ancestry [30]. As a caveat, we reiterate that the choice of the standard sequence was based on the centrality and overall predominance of the sequence in each arm of the *S*. *guianense* complex, rather than on synapomorphic rearrangements for which polarity will need to be determined once appropriate outgroups are examined [31].

We used all inversions shared between two or more populations to produce a character (inversion) matrix for the 25 populations. However, IIL-14, which was found in a single larva in each of two geographically distant populations (Branco and Jacareí) is considered a misread in one of the populations and was not used in the character matrix for our analysis. Four character states were proposed for the inversions: 0 = absent, 1 = fixed, 2 = autosomally polymorphic, and 3 = X-linked.

The matrix was constructed using the software WinClada [32] and was exported to TNT [33,34], where searches were conducted under implied weights with different values of *k* (i.e., 2, 3, 4, 5, 7, and 10). This procedure was performed to evaluate potential topological changes under different *k* values [35]. Searches were conducted using the traditional search command (under Analyze), with 10,000 replications of random addition sequences, followed by tree bisection and reconnection, saving 100 optimal trees per replication. Relative Bremer support [36] was calculated as a measure of group support, using 1000 suboptimal trees up to five steps longer than the shortest tree (obtained using a traditional search, under *k* = 5). The trees were rooted using cytoform C, a monomorphic segregate with a banding sequence identical to the standard sequence for *S*. *guianense s*. *l*.

## Results

### Chromosomal generalities

Of 616 larvae of *S*. *guianense s*. *l*. prepared for analysis, 607 (98.5%) were scored for the entire banding sequence; the 9 partially scored or unscored larvae were not used in any analyses. The typical haploid complement of three submetacentric chromosomes was preserved across all populations. The nucleolar organizer was in the base of IL (section 24/25 junction). A chromocenter and supernumerary (B) chromosomes were absent.

### Chromosomal groups and cytoforms

**Fig 4:**
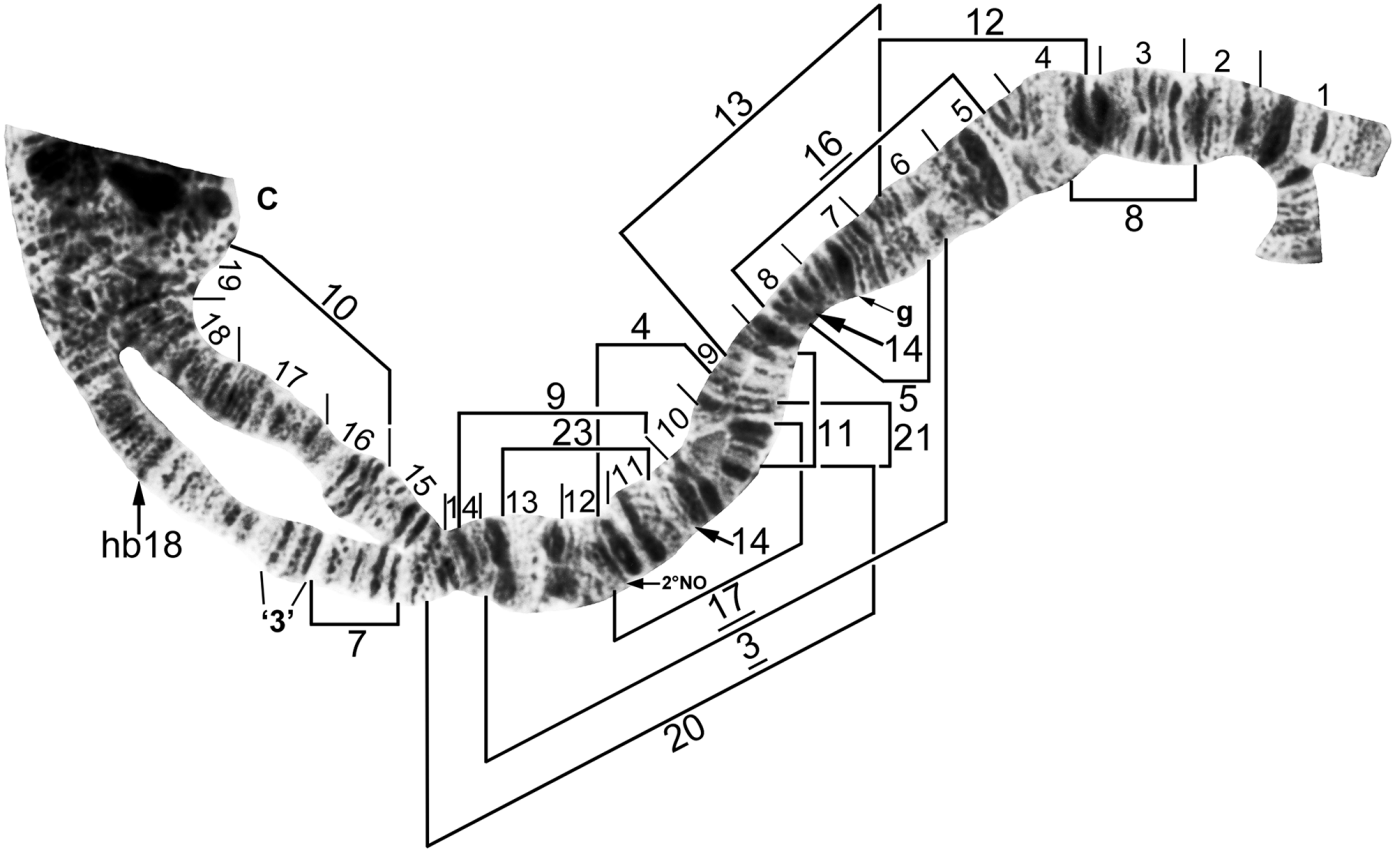
IS standard chromosomal sequence of cytoform F. Breakpoints of 15 inversions of various cytoforms are indicated by brackets, and those of X-linked IS-14, which occurs only on top of IS-13 in the Pitinga River population of cytoform B2, by arrows. Male larva (Longá River). C = centromere, g = glazed marker, hb18 = location of heteroband, 2°NO = location of secondary nucleolar organizer, ‘3’ = 3 heavy marker.

**Fig 5:**
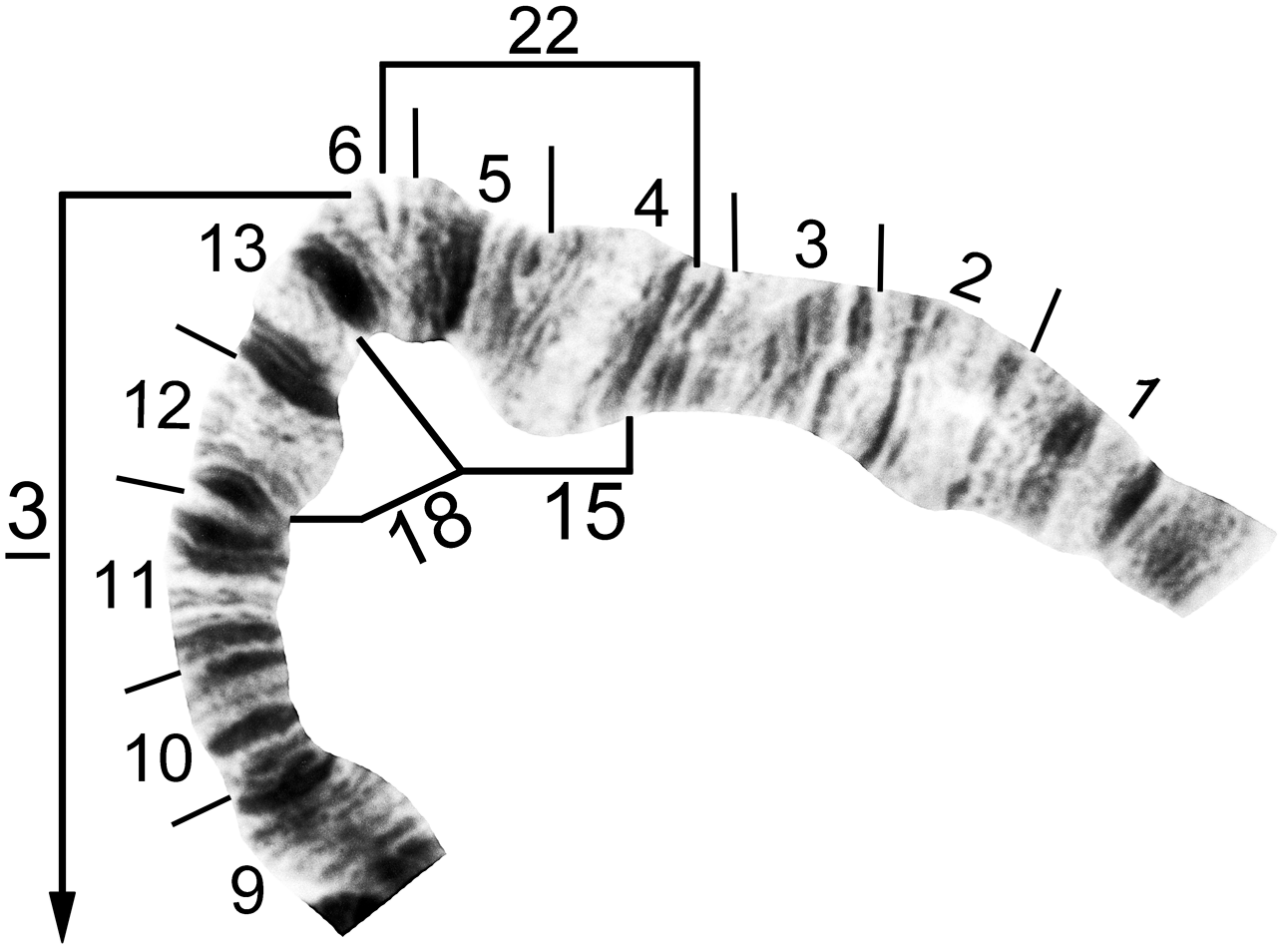
IS distal half of cytoform B3, showing a partial *IS-3* chromosomal sequence. Breakpoints of floating inversions IS-15, IS-18, and IS-22 are indicated by brackets. Male larva (Oiapoque River).

**Fig 6:**
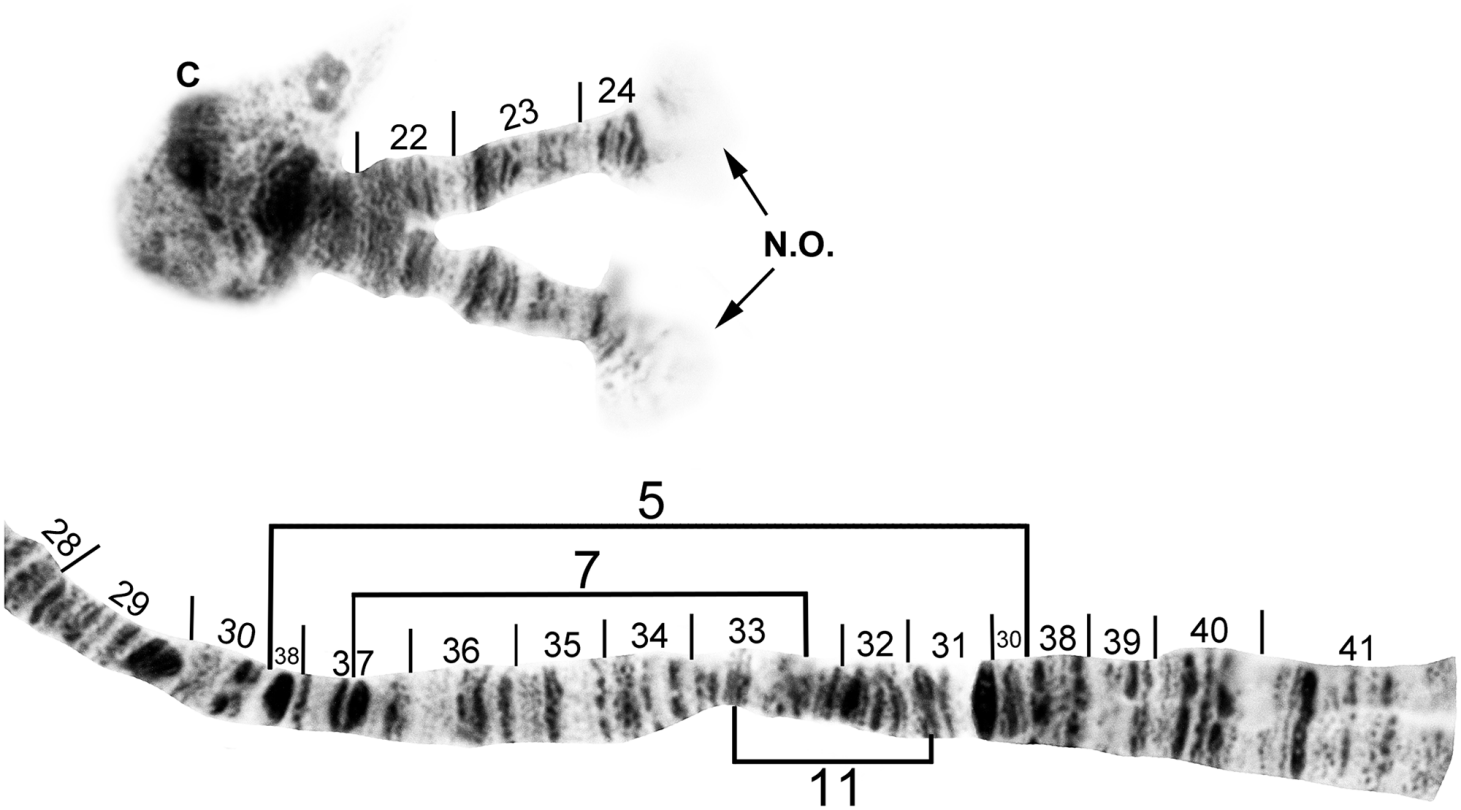
IL base (above) and sections 28–41 of cytoform F, showing IL-5 chromosomal sequence. Breakpoints of IL-7 and IL-11 are indicated by brackets. Male larva (Longá River). C = centromere, N.O. = nucleolar organizer.

**Fig 7:**
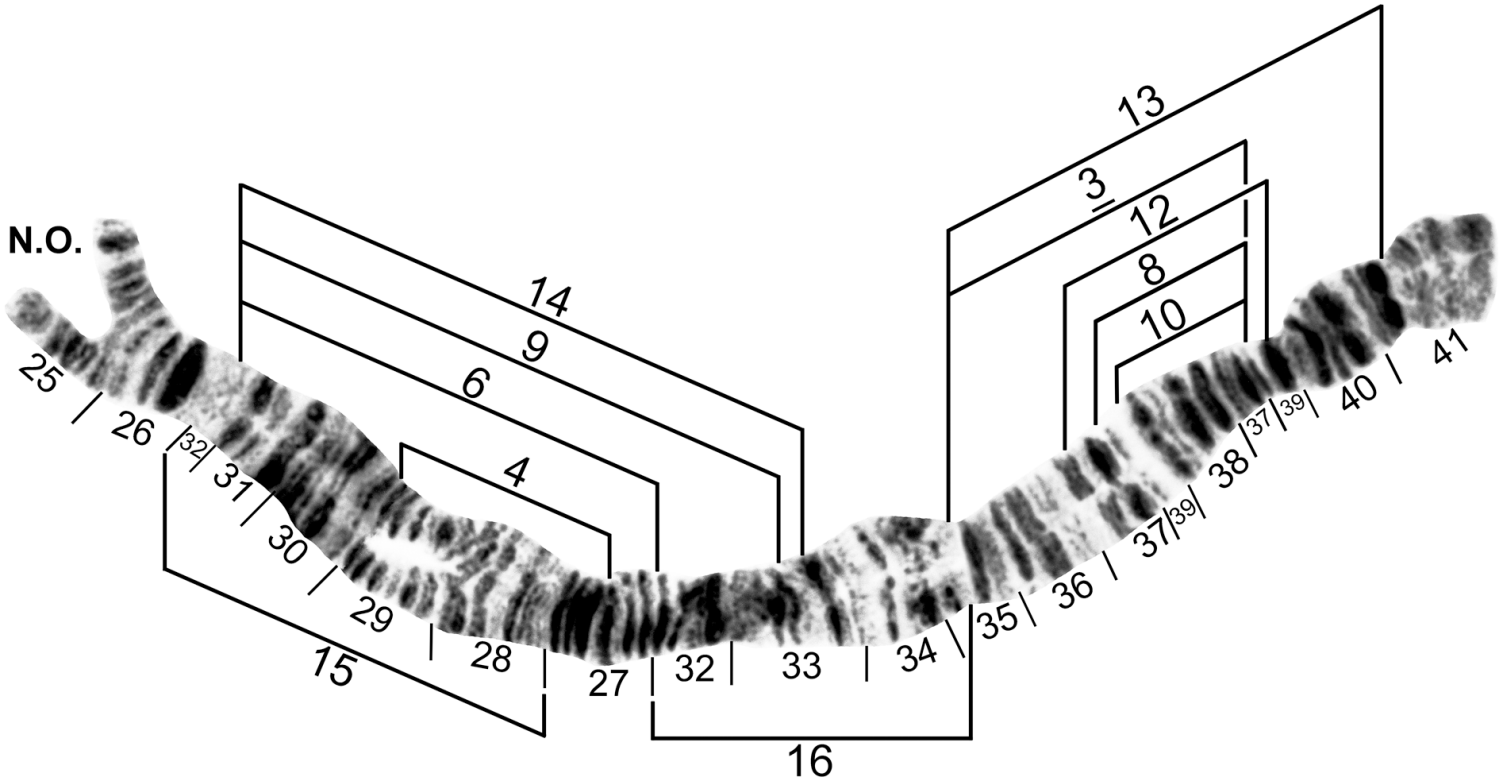
IL distal to nucleolar organizer (N.O.) of cytoform B3, showing IL-6, 8 chromosomal sequence. Breakpoints of 9 additional inversions are indicated by brackets. Male larva (Oiapoque River).

**Fig 8:**
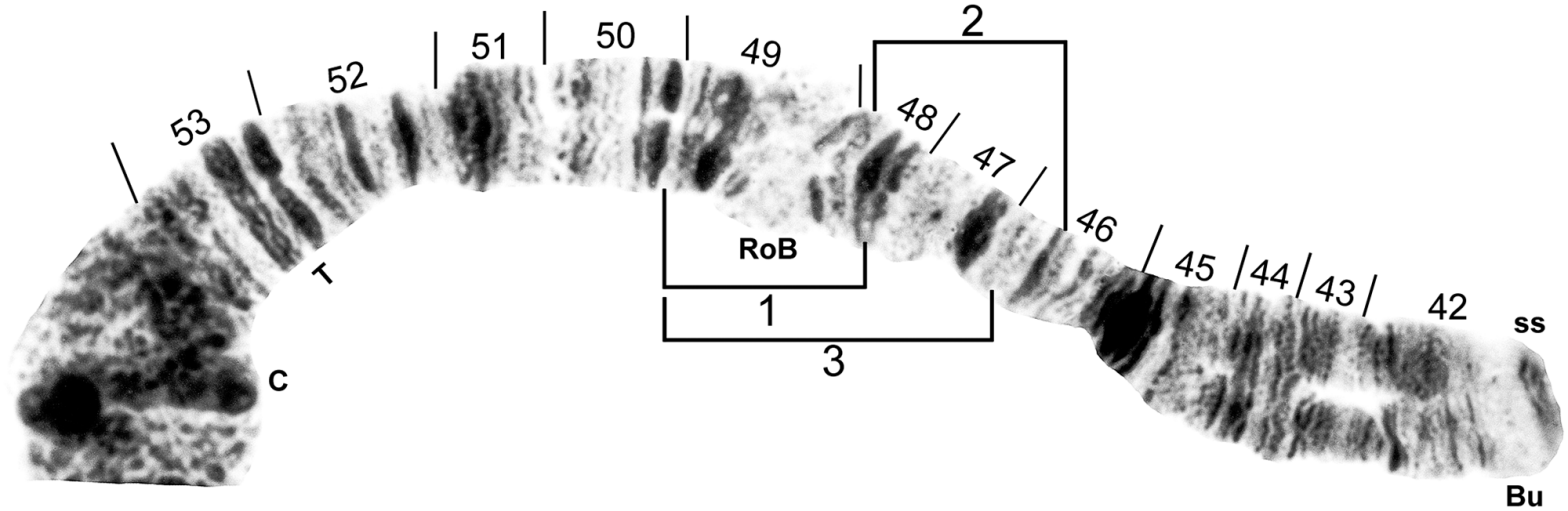
IIS standard chromosomal sequence of cytoform F. Breakpoints of IIS-1, IIS-2, and IIS-3 are indicated by brackets. Composite female [sections 42–45] and male larva [sections 46–54] (Longá River). Bu = bulge, C = centromere, RoB = ring of Balbiani, ss = shoestring marker, T = trapezoidal marker.

**Fig 9:**
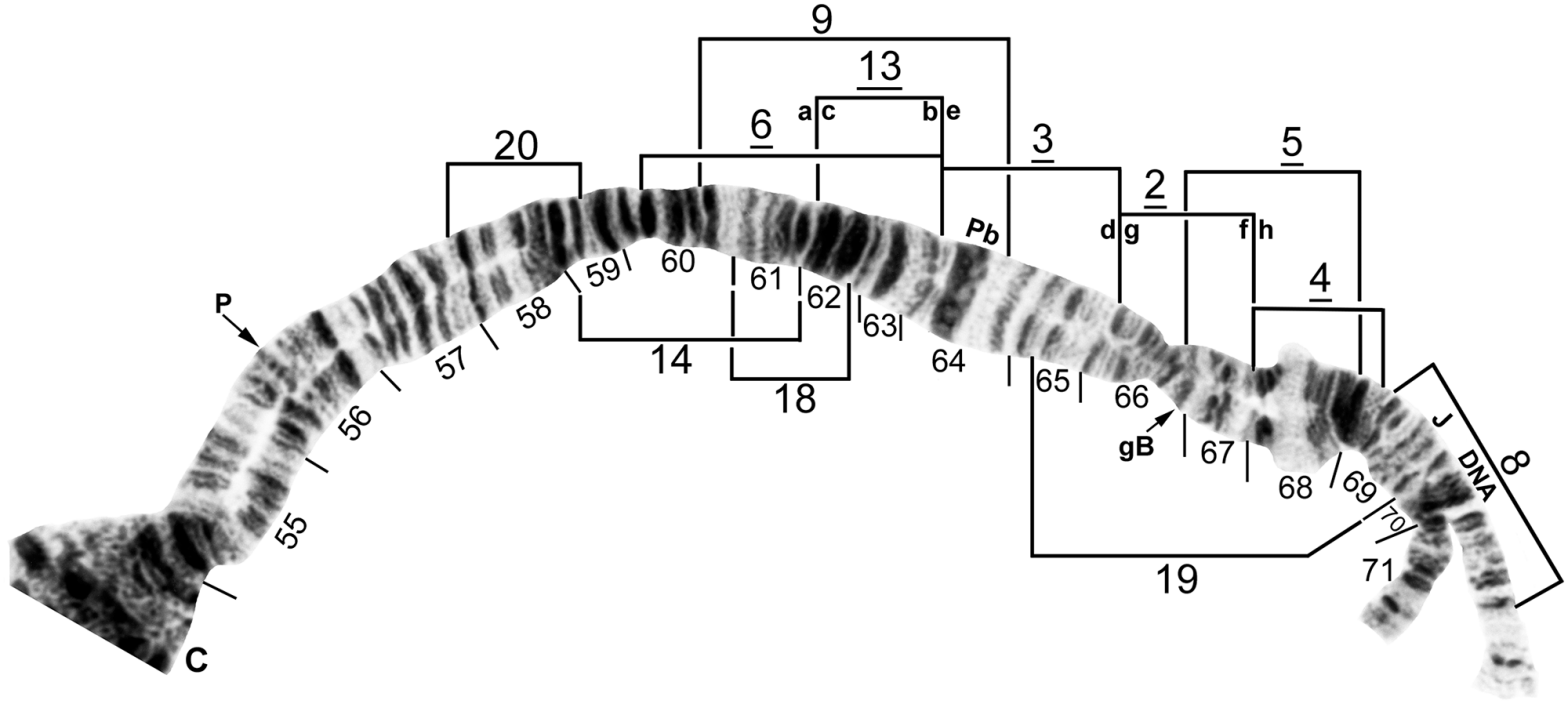
IIL standard chromosomal sequence of cytoform F. Breakpoints of 12 inversions of various cytoforms are indicated by brackets. Letters a—h, when assembled alphabetically, produce the *IIL-2*, *3*, *13* sequence of cytoform B1 from the standard sequence. Male larva (Longá River). C = centromere, gB = gray band, J = jagged, P = puffing band, Pb = parabalbiani.

**Fig 10:**
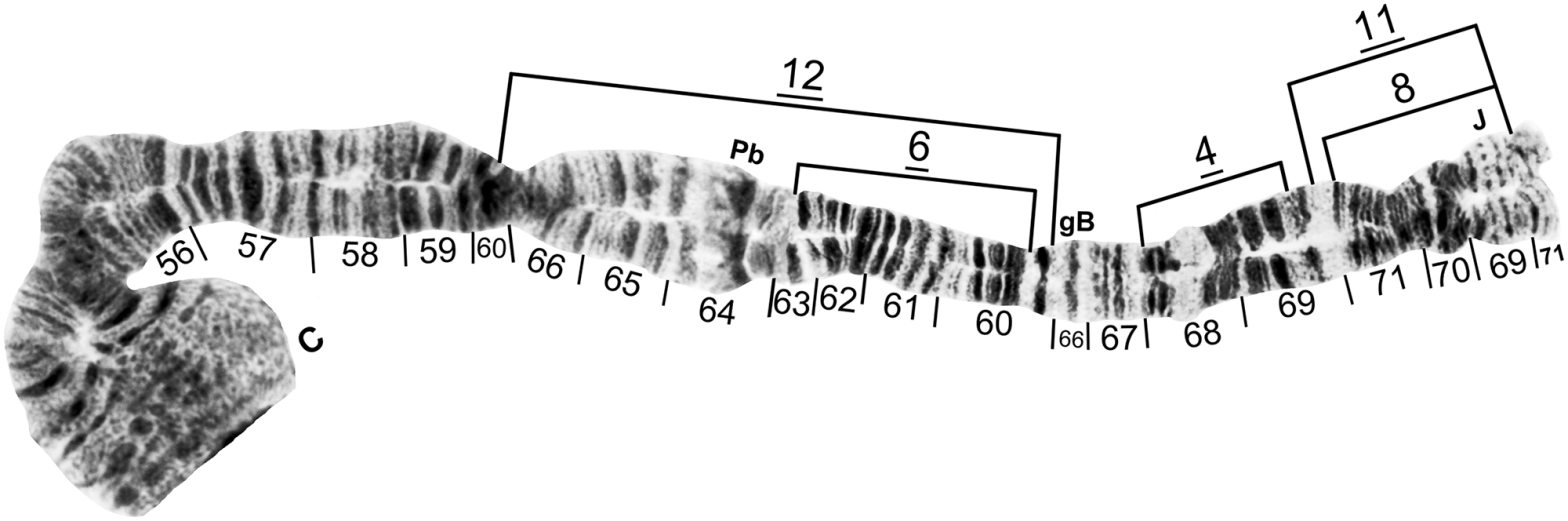
IIL of cytoform E4, showing the *IIL-11*, *12* chromosomal sequence. Breakpoints of *IIL-4*, *IIL-6*, and IIL-8 are indicated by brackets. Female larva (Verdão River). C = centromere, gB = gray band, J = jagged, Pb = parabalbiani.

**Fig 11:**
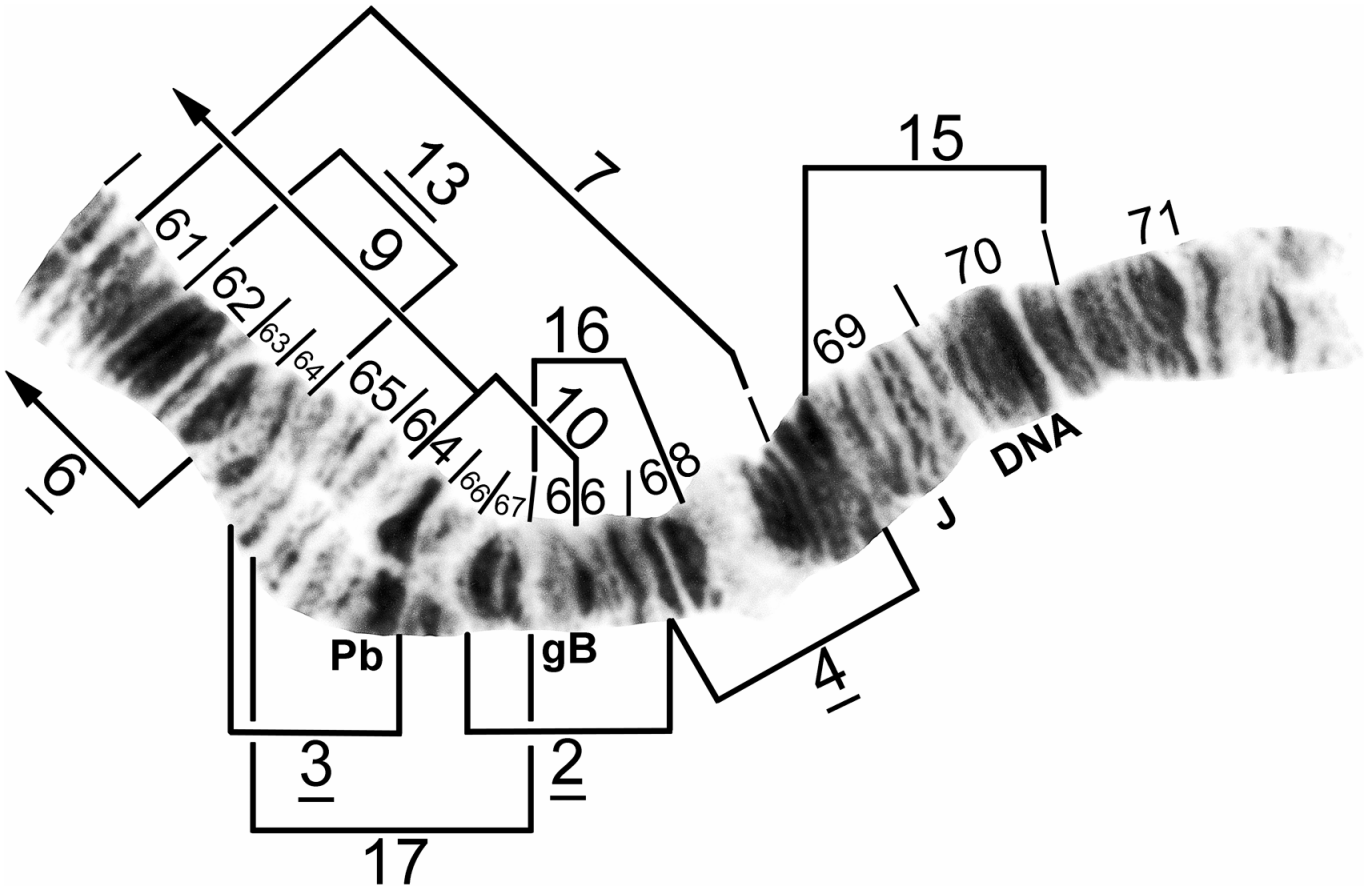
IIL distal half of cytoform B3, showing the *IIL-2*, *3* chromosomal sequence. Breakpoints of 9 additional inversions of various cytoforms are indicated by brackets. Male larva (Oiapoque River). DNA = DNA puff, gB = gray band, J = jagged, Pb = parabalbiani.

**Fig 12:**
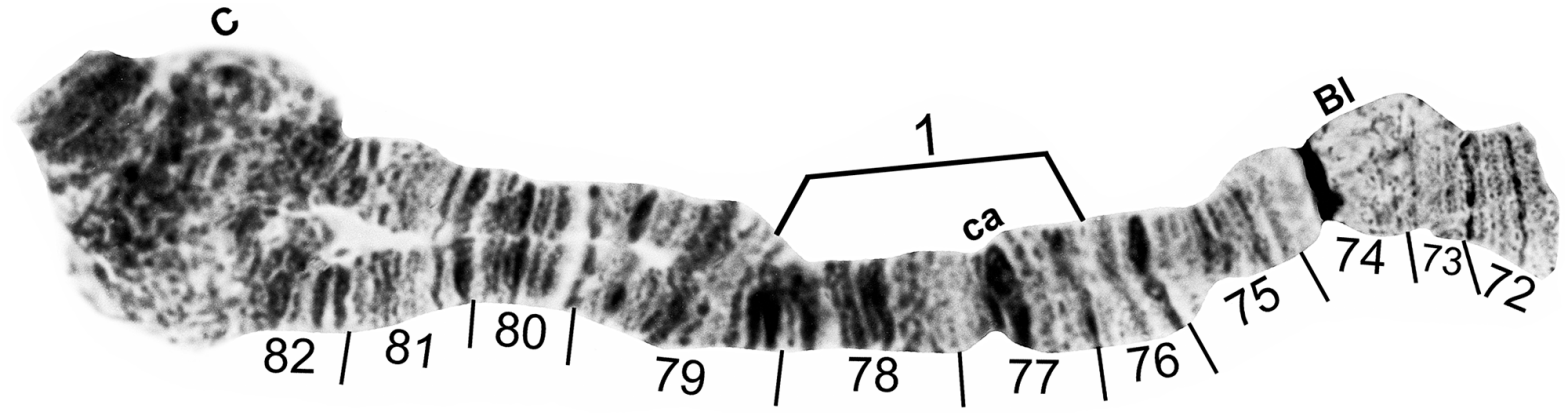
IIIS standard chromosomal sequence of cytoform B3. Breakpoints of IIIS-1 are indicated by a bracket. Male larva (Oiapoque River). Bl = blister, C = centromere, ca = capsule.

**Fig 13:**
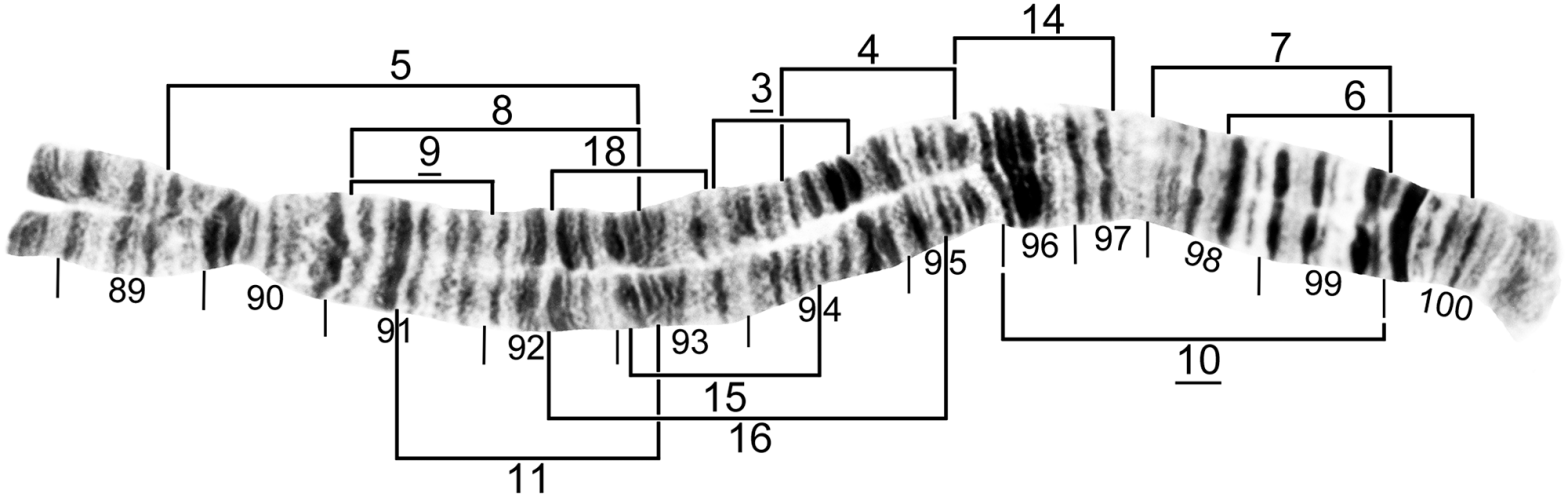
IIIL distal to section 88 of cytoform F, showing standard chromosomal sequence. Breakpoints of 13 inversions of various cytoforms are indicated by brackets. Male larva (Longá River).

**Fig 14:**
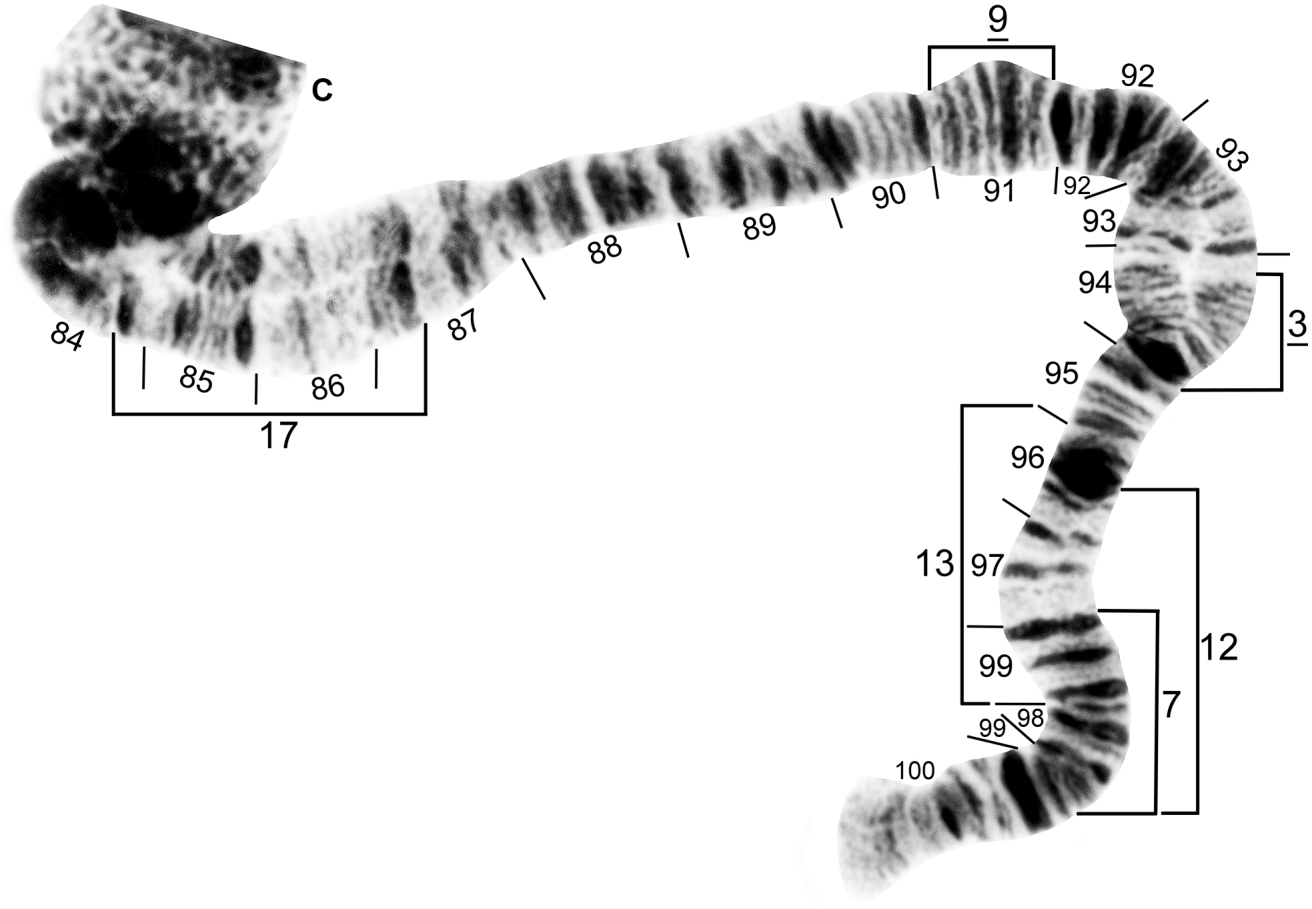
IIIL of cytoform B3, showing IIIL-7, *9* chromosomal sequence. Breakpoints of 4 additional inversions of various cytoforms are indicated by brackets. Female larva (Oiapoque River). C = centromere.

We recorded 83 chromosomal rearrangements, including 81 paracentric inversions, 1 heteroband, and 1 secondary nucleolar organizer, among 607 larvae in 25 populations of *S*. *guianense s*. *l*. Inversions (fixed and polymorphic) were distributed in approximately equal numbers in IS and the 3 long arms, but were scarce in IIS and IIIS: 23 inversions in IS, 16 in IL, 3 in IIS, 20 in IIL, 1 in IIIS, and 18 in IIIL. Average inversion heterozygosity per larva ranged from 0.00 in the monomorphic Farinha River and Teles Pires River populations to 2.92 in the Ereu River population. We recognize the following three chromosomal groups and 13 cytoforms whose diagnostic chromosomal rearrangements are summarized in Table 2 and Figs 4–14:

**Table 2:** Overview of diagnostic chromosomal rearrangements of 13 cytoforms of *Simulium guianense s*. *l*.

Cytoform	Fixed inversions	Common^1^ autosomal polymorphisms	Sex chromosomes^2^
A1	*IIL-4*, *IIL-6*, *IIIL-3*	none > 0.20	X_0_Y_0_
A2	*IL-8*, *IIL-4*, *IIL-6*	IIIL-3	X_0_Y_0_
B1	*IIL*-2, *IIL*-3, *IIL*-13, *IIIL*-7, *IIIL*-9	none > 0.05	X_0_Y_0_
B2	*IS*-3, *IIL*-2, *IIL*-3, *IIIL*-9	IL-6, IL-8, IIIL-7	X_0_X_0_, X_0_X_1_, X_1_X_1_, X_1_X_2_, X_3_X_3_, X_0_Y_0_, X_1_Y_0_, X_1_Y_1_, X_3_Y_0_
B3	*IS*-3, *IL*-6, *IIL*-2, *IIL*-3, *IIIL*-9	IL-8, IIIL-7	X_0_Y_0_
B4	*IS*-3, *IL*-6, *IIL*-2, *IIL*-3, *IIIL*-9	IL-4, IIS-1, IIL-8, IIL-9, IIIL-5	X_0_X_0_, X_0_X_1_, X_1_X_1_, X_0_Y_0_, X_1_Y_0_, X_1_Y_1_
C	standard sequence	none (monomorphic)	X_0_Y_0_
D	*IS-16*, *IS-17*, *IL-3*, *IIL-5*	IIL-18	X_0_Y_0_
E1	*IIS-2*	none > 0.15	X_0_Y_0_
E2	*IS-12*, *IIIL-10*	IL-8, IL-11, IIS-2	X_0_Y_0_
E3	standard sequence	IL-8, IIS-2, IIIL-11	X_0_Y_0_
E4	*IIL-11*, *IIL-12*	IS-12, IL-9, IL-10, IIS-2	X_0_Y_0_
F	standard sequence	IL-5	X_0_Y_0_

^1^ Represented in at least half the populations of a cytoform at a frequency ≥ 0.20 per population.

^2^ Details of differentiated sex chromosomes are given under respective cytoform treatments.

### Group A

Two cytoforms, one in the Central-West Region and the other in the Southeast Region comprised the A group. Our reinterpretation of the chromosomal photomaps of Charalambous et al. [17] depicted the A group as uniquely defined by *IIL-4* and *IIL-6* (Figs 9–11).

#### Cytoform A1

We had no samples of cytoform A1, as described by Charalambous et al. [17] from two sites in the Toncantins-Araguaia ecoregion of Brazil. Our data were drawn from our reinterpretation of the information provided by Charalambous et al. [17]. A1 was fixed for *IIIL-3* (Figs 13 and 14). Five low-frequency (< 0.16) polymorphic inversions and cytologically undifferentiated sex chromosomes were recorded by Charalambous et al. [17] (Table 3).

**Table 3:** Frequency of inverted chromosomal constituents for *Simulium guianense* cytoforms A1, A2, C, D, and F.

Cytoform	A1^1^	A2	C	D	D	F	F	F
River	Toncantins	Pandeiros	Farinha^2^	Xingu	Iriri	Jacareí	Pirangi	Longá
♀:♂	NA	8:13	22:10	29:13	3:6	6:5	4:6	21:8
IS-1	0.09							
IS-2	0.06							
IS-10								0.02
*IS-16*				1.00	1.00			
*IS-17*				1.00	1.00			
IS-23		0.10						
IL-1	0.16							
*IL-3*				1.00	1.00			
IL-4								
IL-5						0.73	0.30	0.83
*IL-8*		1.00						
IL-16		0.14						
IIL-1	0.04							
*IIL-4*^3^	1.00	1.00						
*IIL-5*				1.00	1.00			
*IIL-6*^3^	1.00	1.00						
IIL-18				1.00	0.89			
IIIL-1	0.03							
IIIL-3^3^	1.00	0.43						
IIIL-6						0.23	0.05	0.10
IIIL-14						0.05		
IIIL-15				0.01				
IIIL-18				0.01				
Heter.^4^	NA	1.24	0.00	0.05	0.22	0.73	0.50	0.52

^1^ All data from Charalambous et al. [17] for two populations combined (Toncantins and Mucambão rivers); sex ratio and number of heterozygotes per larva not given (NA), but 34–36 larvae were scored for the listed inversions.

^2^ Years 2000 (*n* = 23) and 2001 (*n* = 9) combined.

^3^ IIL-4, IIL-6, and IIIL-3 are shown as homozygous standard by Charalambous et al. [17] in their figures (Figures 5 and 6 in [17]) and as standard in all larvae in their Minaçu (cytoform A1) populations. However, we consider these sequences to be inverted in the Minaçu (cytoform A1) populations because they have the opposite polarity, i.e., the inverted sequence *sensu* Charalambous et al. [17], in all other studied populations (except the closely related cytoform A2).

^4^ Average number of heterozygous autosomal inversions per larva.

#### Cytoform A2

Our population of this cytoform from the state of Minas Gerais, Brazil, in the São Francisco ecoregion differed from A1 by having *IL-8* fixed (Fig 7), which it shared with some members of the B group, and IIIL-3 as an autosomal polymorphism (frequency = 0.43). It had two additional, unique polymorphisms (IS-23 and IL-16; Figs 4 and 7) with frequencies less than 0.15 (Table 3). The homologues were loosely paired, and the sex chromosomes were cytologically undifferentiated.

### Group B

The B group, a cluster of four cytoforms, was uniquely defined by *IIL*-2, *IIL*-3, and *IIIL*-9 (Table 4; Figs 9, 11 and 14). All but B1 also shared *IS-3* (Figs 4 and 5) and IL-6 (Fig 7). B2, B3, and B4 represented all of our populations in Brazil’s North Region, French Guiana, and southeastern Venezuela. B1 in the Teles Pires River of the Central-West Region, was the most geographically remote member of the B group, almost 1500 km from the nearest other population (Pitinga River) in the B group. IIIL-7 (Fig 14) was fixed in all B group members except B4 and the Oiapoque and Maroni populations of B2.

**Table 4:** Frequency of inverted chromosomal constituents for group B of *Simulium guianense s*. *l*.

Cytoform	B1	B2	B2	B2	B2	B2	B2	B2	B3	B3	B4	B4
River	Teles Pires	Branco	Água Fria	Pitinga	Caroebe	Ereu	Cachorro	Maroni	Tracajatuba	Oiapoque	Parupa	Yuruani
♀:♂	12:11	4:3	3:5	17:14	16:9	11:13	18:9	10:12	9:9	28:35	22:16	7:7
*IS-3*		1.00	1.00	1.00	1.00	1.00	1.00	1.00	1.00	1.00	1.00	1.00
IS-4												0.04
IS-5										0.01		
IS-6^1^												
IS-7												0.04
IS-8		0.07										*^2^
IS-9												0.18
IS-11										0.01		
IS-13		0.50	0.31	****^5^	**^3^	***^4^	**^3^	0.02				
IS-14				****^5^								
IS-15		0.21				***^4^						
IS-18											0.04	
IS-20								0.02				
IS-21								0.02				
IS-22								0.02				
IL-2^1^												
IL-4											0.01	0.25
IL-5												
IL-6		0.21	1.00	1.00	1.00	1.00	1.00	1.00	1.00	1.00	1.00	1.00
IL-8			0.75	0.64^7^	0.24		0.15	0.62	0.97	0.85		
IL-12						0.98						
IL-13						0.96						
IL-14		*****^6^										
IL-15		0.07										
IL-16												
IIS-1											0.01	0.29
IIS-3					0.18		0.04					
*IIL-2*	1.00	1.00	1.00	1.00	1.00	1.00	1.00	1.00	1.00	1.00	1.00	1.00
*IIL-3*	1.00	1.00	1.00	1.00	1.00	1.00	1.00	1.00	1.00	1.00	1.00	1.00
IIL-7												0.07
IIL-8											0.20	
IIL-9											0.45^7^	
IIL-10								0.02		0.01		0.04
*IIL-13*	1.00											
IIL-14				0.31	0.12	0.56^7^						
IIL-15						0.06						
IIL-16						0.52^7^						
IIL-17						0.50^7^						
IIL-19								0.12				
IIL-20								0.26				
IIIS-1						0.19						
IIIL-2^1^												
IIIL-4												0.18
IIIL-5												0.29
IIIL-7	1.00	1.00	1.00	1.00	1.00	1.00	1.00	0.88	1.00	0.91		
*IIIL-9*	1.00	1.00	1.00	1.00	1.00	1.00	1.00	1.00	1.00	1.00	1.00	1.00
IIIL-12			0.19		0.04		0.02	0.14				
IIIL-13						0.50^7^						
IIIL-14		0.14										
IIIL-17											0.01	
Heter.^8^	0.00	2.57	1.00	0.81	0.92	2.92	0.41	1.77	0.06	0.49	0.92	2.79

^1^ IS-6, IL-2, and IIIL-2 were found in low frequency (< 0.10) in the Oiapoque population by Charalambous et al. [17]; we did not find these inversions in our material.

^2^ * Possible linkage of IS-8 to the X chromosome (X_0_ and Y_0_ = undifferentiated, X_1_ and Y_1_ = IS-8): 1 X_0_X_0_, 1 X_0_X_1_, 5 X_1_X_1_, 1 X_0_Y_0_, 5 X_1_Y_0_, and 1 X_1_Y_1_.

^3^ ** Absolute linkage of IS-13 to the X chromosome (Table 5).

^4^ *** Partial linkage of IS-13 to the X chromosome; 1 of these males was heterozygous for IS-13 and IS-15 (Table 5).

^5^ **** Partial linkage of IS-13 to the X chromosome; IS-14 was linked to the X in 2 of these individuals (Table 5).

^6^ ***** Possible linkage of IL-14 to the X chromosome (Table 5).

^7^ Tested for Hardy-Weinberg equilibrium; all were in equilibrium (df = 1, P > 0.05).

^8^ Average number of heterozygous autosomal inversions per larva.

#### Cytoform B1

Though lacking *IS-3*, cytoform B1 from Brazil’s Teles Pires River in the Tapajós-Juruena ecoregion joined the B group by sharing *IIL-2*, *IIL-3*, and *IIIL-9* (Table 4; Figs 11 and 14). It was fixed for *IIIL-7* and uniquely fixed for *IIL-13* (Figs 9 and 14). The only polymorphisms were a secondary nucleolar organizer heterozygously expressed in section 12 of one female larva and a heteroband in section 18 of two male larvae (Fig 4). Sex chromosomes were cytologically undifferentiated.

#### Cytoform B2

This cytoform included six populations from the Amazonas Guiana Shield ecoregion and one from the adjacent Guianas ecoregion, all of which shared IS-13 (Table 4; Fig 4), in addition to *IS-3*, IL-6, *IIL-2*, *IIL-3*, IIIL-7, and *IIIL-9*. IS-13, however, was rare (frequency = 0.02) in the Maroni River population, and, if missed, might have led to assignment of this population to cytoform B3. Four of the seven populations of cytoform B2 had at least partial linkage of IS-13 to the X chromosome (Table 5). Larger samples from the Branco and Água Fria rivers might have revealed partial X linkage of IS-13. If, however, IS-13 is strictly autosomal in the Branco and Água Fria populations, they could join B3, the implication being that IS-13 was lost in the B3 populations of the Oiapoque and Tracajatuba rivers. Alternatively, the apparent linkage of IL-14 (Fig 7) to the X in the Branco River population, if upheld by larger samples, would suggest another cytoform in the B group. Of 24 different autosomal polymorphisms in populations of B2, half had frequencies greater than 0.20 in at least one population (Table 4, Figs 4, 5, 7, 9, 11 and 14): IS-13, IS-15, and IL-6 in the Branco River; IS-13 and IL-8 in the Água Fria River; IL-8 and IIL-14 in the Pitinga River; IL-8 in the Caroeobe River; IL-12, IL-13, IIL-14, IIL-16, IIL-17, and IIIL-13 in the Ereu River; and IL-8, IIL-20, and IIIL-7 in the Maroni River. The centromeres paired ectopically in at least some nuclei of 12% of the Ereu population. The chromosomes of the Branco population were more condensed than those of any other population. IL-8 in the Pitinga River was in Hardy Weinberg equilibrium (12 ss, 12 si, 7 ii; df = 1, χ^2^ = 1.30, P > 0.05), as were the following four inversions in the Ereu River (df = 1, P > 0.05): IIL-14 (4 ss, 13 si, 7 ii; χ^2^ = 0.24), IIL-16 (5 ss, 13 si, 6 ii; χ^2^ = 0.17), IIL-17 (5 ss, 14 si, 5 ii; χ^2^ = 0.67), and IIIL-13 (5 ss, 14 si, 5 ii; χ^2^ = 0.67) (Table 4).

**Table 5:** Sex chromosomes in populations of *Simulium guianense* cytoform B2.

Population	Sex Chromosomes^1^								
	X_0_X_0_	X_0_X_1_	X_1_X_1_	X_1_X_2_	X_3_X_3_	X_0_Y_0_	X_1_Y_0_	X_1_Y_1_	X_3_Y_0_
Água Fria	3	0	0	0	0	5	0	0	0
Branco	1	0	0	0	3	0	0	0	3
Cachoro	0	0	18	0	0	0	9	0	0
Caroebe	0	0	16	0	0	0	9	0	0
Ereu	0	0	11	0	0	1	7^2^	5	0
Maroni	10	0	0	0	0	12	0	0	0
Pitinga	0	6	9	2	0	1	12	1	0

^1^ X_0_ and Y_0_ = microscopically undifferentiated, X_1_ = IS-13, X_2_ = IS-13+14, X_3_ = IL-14, Y_1_ = IS-13.

^2^ 1 X_1_Y_0_ male was also heterozygous for IS-15, but whether IS-15 was associated with the X or the Y could not be determined.

#### Cytoform B3

This cytoform was represented by one population from the Oiapoque Basin (Guianas ecoregion) and one from the Araguari Basin (Amazonas Estuary & Coastal Drainages ecoregion) in Brazil. In addition to *IS-3*, *IL-6*, IL-8, *IIL-2*, *IIL-3*, IIIL-7, and *IIIL-9*, the two populations were weakly united by the absence of IS-13 (Table 4). IL-8 (Fig 7) was in high frequency (> 0.84), and the sex chromosomes were cytologically undifferentiated. IS-5, IS-11, and IIL-10 were rare (0.01) polymorphisms in the Oiapoque population (Figs 4 and 11). Lack of a uniquely shared chromosomal character prohibited us from including the Oiapoque and Tracajatuba populations in any other cytoform in the B group. We did not find 3 low-frequency (< 0.10) inversions (IS-6, IL-2, and IIIL-2) discovered in the Oiapoque population by Charalambous et al. [17]. The absence of IL-8 and IIIL-7 in the Oiapoque population sampled 20 years earlier about 3 km from our collection site and studied by Charalambous et al. [17] could not be reconciled with the high frequency of these inversions (0.85 and 0.91, respectively) in our material.

#### Cytoform B4

The two Venezuelan populations from the Orinoco Guiana Shield ecoregion had *IS-3*, *IL-6*, *IIL-2*, *IIL-3*, and *IIIL-9*. They were united, albeit weakly, by floating inversions IL-4 and IIS-1 (Figs 7 and 8) and absence of IIIL-7, which was fixed, or nearly so, in all other members of the B group. IS-8 possibly was linked to the X chromosome in the Yuruani River population (Table 4, Fig 4). Of 12 additional autosomal polymorphisms, only IIL-8 and IIL-9 (Fig 9) in the Parupa River population and IL-4, IIS-1, and IIIL-5 (Figs 7, 8 and 13) in the Yuruani population had frequencies of 0.20 or higher (Table 4). IIL-9 (12 ss, 18 si, 8 ii) in the Parupa population was in Hardy-Weinberg equilibrium (df = 1, χ^2^ = 0.07, P > 0.05).

### Cytoform C

A population from the Farinha River in the Toncantins-Araguaia ecoregion of Brazil represented cytoform C, a monomorphic segregate in our study (Table 3). Its banding sequence was identical to the standard for *S*. *guianense s*. *l*. Our results agreed with those of Charalambous et al. [17], who found that the Toncantins River population about 30 km downriver from our site, a tributary of the Tocantins River, was monomorphic. *IIL-4* and *IIIL-3* of Charalambous et al. [17] are actually standard in this cytoform, as depicted on our modified standard map (Figs 9 and 13). Sex chromosomes were microscopically undifferentiated.

### Cytoform D

Two populations in Brazil’s Xingu ecoregion represented cytoform D. They uniquely shared fixed inversions *IS-16*, *IS-17*, *IL-3*, and *IIL-5*, plus IIL-18 (Figs 4, 7 and 9), which was fixed in the Xingu River and in high frequency (0.89) in its tributary, the Iriri River (Table 3). The only other rearrangements were IIIL-15 and IIIL-18 (Fig 13), which appeared heterozygously in one larva each in the Xingu River. Sex chromosomes were cytologically undifferentiated. The chromosomal homologues of both populations were the most loosely paired among all of our samples. IIL-18 appears to be present on the map of Charalambous et al. (Figure 19 in [17]), though overlooked. The putative absence of *IS-16* and *IS-17* reported for the Xingu population by Charalambous et al. [17] could not be reconciled with our population analysis. We note, however, that the two populations were sampled about 14 years and 20 km (straight line) apart.

### Group E

Five populations along the southern margin of the distribution of *S*. *guianense s*. *l*., in the Central-West and Southeast regions of Brazil, were characterized by a high frequency (0.29–1.00) of IIS-2 (Fig 8). Sex chromosomes were cytologically undifferentiated for all group members. The presence of three inversions (IL-6, IL-8, and IIIL-7) in at least one member of the E group (Table 6), which also were shared with the B group, suggested a relationship between the B and E groups; the inference is that these inversions were polymorphic in a hypothetical ancestor of the two groups.

**Table 6:** Frequency of inverted chromosomal constituents for group E of *Simulium guianense s*. *l*.

Cytoform	E1	E2	E3	E3	E4
River	São José	Araguaia	Mortes	Pardo	Verdão
♀:♂	19:11	22:23	6:9	14:6	25:18
IS-12		1.00			0.24
IS-19^1^				0.02	
IL-6				0.12	
IL-7	0.13				
IL-8		0.40^2^	0.27	0.05	
IL-9					0.57^2^
IL-10					0.21^2^
IL-11		0.22^2^			
IIS-2	1.00	0.29^2^	0.40	0.45	0.90
*IIL-11*					1.00
*IIL-12*					1.00
IIIL-7					0.12
IIIL-8					0.15
*IIIL-10*		1.00			
IIIL-11		0.14	0.30	0.65	
IIIL-16	0.02				
Heter.^3^	0.23	1.27	1.53	1.40	1.81

^1^ IS-19 was represented by a knot with exact breakpoints unresolvable but near those of IS-12.

^2^ All inversions tested were in Hardy-Weinberg equilibrium (df = 1, P > 0.05).

^3^ Average number of heterozygous autosomal inversions per larva.

#### Cytoform E1

Fixation of *IIS-2* (Fig 8) characterized this cytoform from Brazil’s São José River in the Doce Basin of Espírito Santo state (Northeastern Mata Atlantic ecoregion). Cytoform E1 otherwise carried two unique polymorphisms, IL-7 and IIIL-16 (Table 6; Figs 6 and 13).

#### Cytoform E2

The Araguaia River population in the Tocantins-Araguaia ecoregion of Brazil was uniquely fixed for *IS-12* and *IIIL-10* (Figs 4 and 13) and was the only cytoform with IL-11 (Fig 6). It shared IL-8 and IIIL-11 with cytoform E3 and IL-8 with some members of Group B (Figs 7 and 13). The following inversions were in Hardy-Weinberg equilibrium (df = 1, P > 0.05): IL-8 (19 ss, 16 si, 10 ii; χ^2^ = 3.02), IL-11 (29 ss, 12 si, 4 ii; χ^2^ = 2.35), and IIS-2 (24 ss, 16 si, 5 ii; χ^2^ = 0.82) (Table 6).

#### Cytoform E3

Two populations in separate ecoregions represented cytoform E3, weakly characterized by an absence of fixed inversions. Polymorphisms included IS-19, IL-6, IL-8, IIS-2, and IIIL-11 in the Pardo River population (Upper Paraná ecoregion) and IL-8, IIS-2, and IIIL-11 in the Mortes River population (Tocantins-Araguaia ecoregion) (Table 6; Figs 7, 8 and 13).

#### Cytoform E4

The Verdão River population (Upper Paraná ecoregion) was defined by two unique fixed inversions (*IIL-11* and *IIL-12*, Fig 10) and three unique polymorphisms, viz. IL-9, IL-10, and IIIL-8 (Table 6; Figs 7 and 13). IL-9 (7 ss, 23 si, 13 ii; χ^2^ = 0.36) and IL-10 (29 ss, 10 si, 4 ii; χ^2^ = 3.80) were in Hardy-Weinberg equilibrium (df = 1, P > 0.05). IS-12 (Fig 4) was shared with E2, and IIIL-7 (Fig 14) was shared with the B group.

### Cytoform F

Three populations in the Northeast Region, all geographically within 120 km of one another in the Parnaíba ecoregion, were united by the unique floating inversions IL-5 (Fig 6) and IIIL-6 (Fig 13). The sex chromosomes were microscopically undifferentiated. Two rare polymorphisms, IS-10 and IIIL-14 (Figs 4 and 13), were found heterozygously in one Longá and one Jacareí larva, respectively (Table 3). We suspect that IIIL-14 in the Jacareí larva was a misread of a mimic inversion in the Branco River population, given the geographic distance (ca. 2,200 km) and absence of other shared rearrangements between the two populations. Cytoform F was superficially similar to cytoform C, the geographically nearest cytoform, about 650 km northeast in the Farinha River; both cytoforms lacked fixed inversions. The absence of IL-5 and IIIL-6 in cytoform C might reflect clinal variation. Treating C and F as a single cytoform, however, forms a potentially unnatural group without a uniquely shared rearrangement.

### Evolutionary relationships

**Fig 15:**
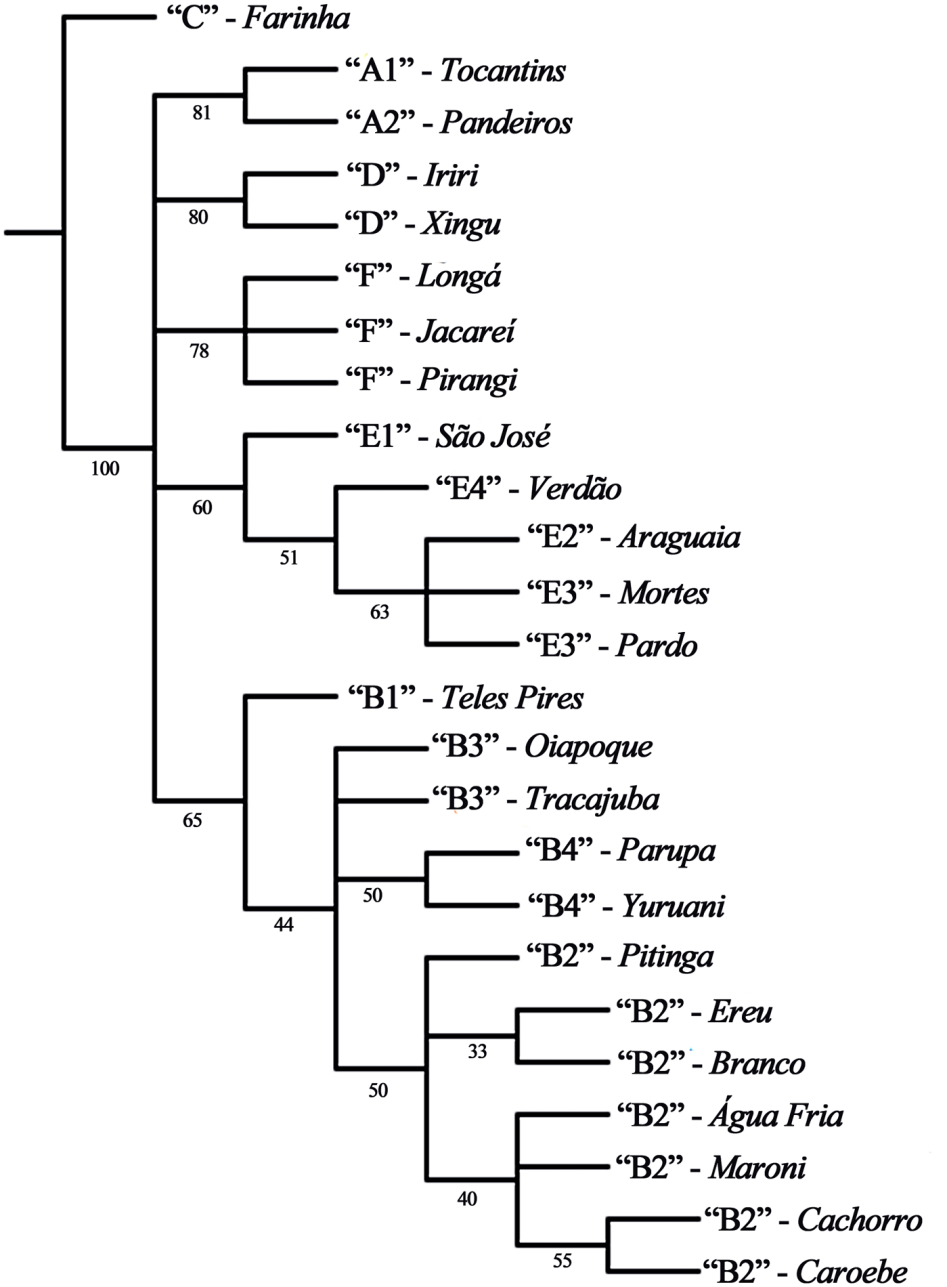
Strict consensus tree of three most parsimonious trees for cytoform populations of *Simulium guianense s*. *l*. Cytoforms are in quotation marks followed by their rivers of occurrence. Numbers below branches correspond to relative Bremer support.

**Fig 16:**
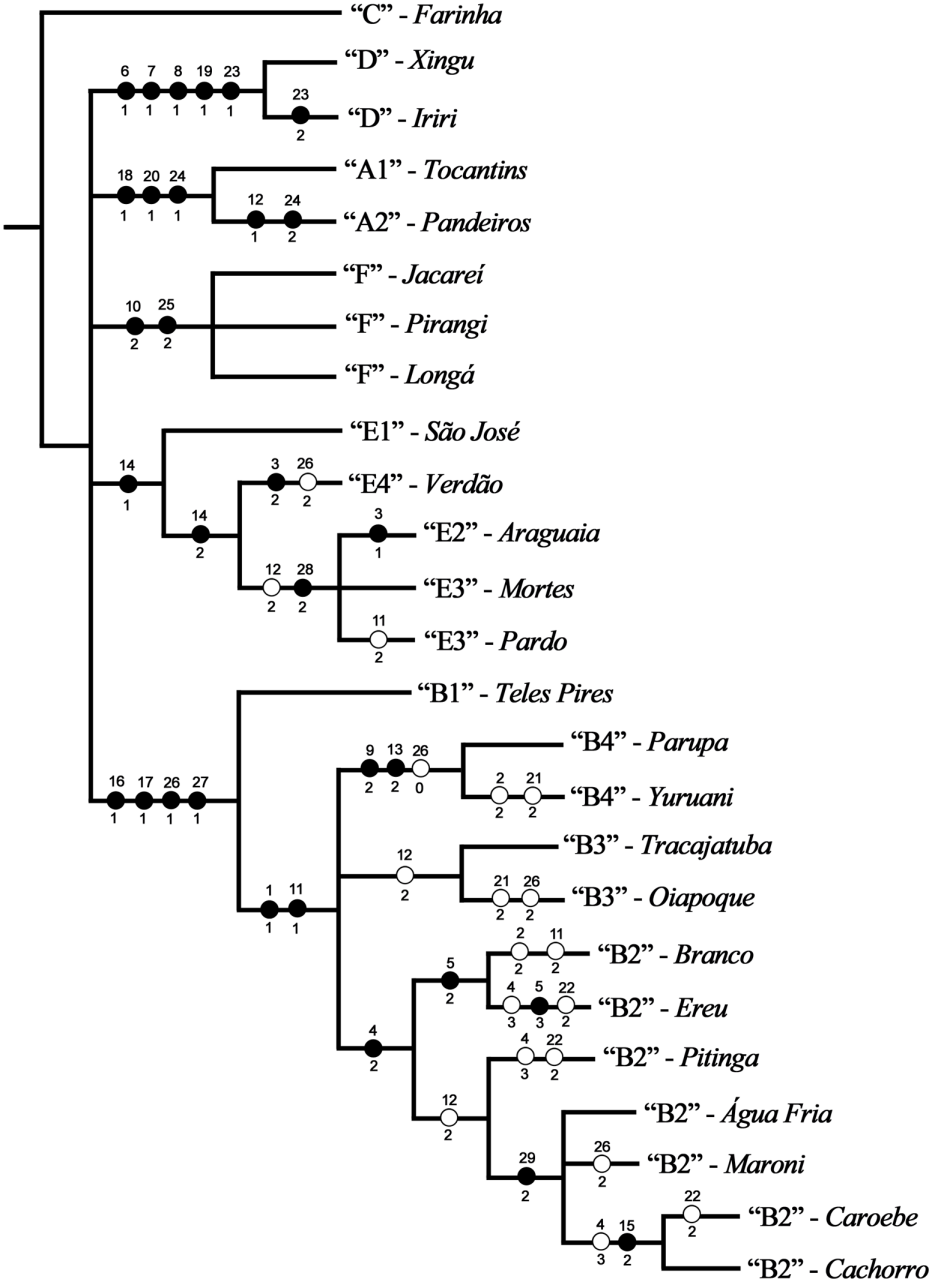
Representative tree showing hypothesis of relationships among cytoform populations of *Simulium guianense s*. *l*. Cytoforms are in quotation marks followed by their rivers of occurrence. Black circles indicate unique apomorphies (i.e. derived traits of a clade); open circles represent non-unique changes on the tree, either forward or reverse.

Our analysis of evolutionary relationships, based on 29 characters (inversions, Table 7), recovered three most parsimonious trees of 72 steps, with consistency and retention indices of 0.69 and 0.85, respectively. The same topologies were achieved for each value of *k* (2, 3, 4, 5, 7, and 10) used in the implied weighted analysis. The basic structure of the three trees was highly similar. All recovered the same major clades (Group A, Group B, Cytoform D, Group E, and Cytoform F) as monophyletic, but in an unresolved polytomy. In addition to the strict consensus tree (Fig 15), we show one of the three trees that provides the greatest resolution of relationships (Fig 16). This tree differed from the other two trees only by 1) grouping the B3 populations of Tracajatuba and Oiapoque rather than placing them separately either in a trichotomy with the B2 cluster or in a polytomy with B4 and members of the B2 cluster, and 2) resolving the placement of Pitinga rather than placing it in a trichotomy with Branco + Ereu and all other B2 populations.

**Table 7:** Data matrix of 29 characters used in cladistic analysis of populations of *Simulium guianense s*. *l*.

Cytoform	Population	Character state^1^																												
		1	2	3	4	5	6	7	8	9	10	11	12	13	14	15	16	17	18	19	20	21	22	23	24	25	26	27	28	29
C	Farinha	0	0	0	0	0	0	0	0	0	0	0	0	0	0	0	0	0	0	0	0	0	0	0	0	0	0	0	0	0
A1	Toncatins	0	0	0	0	0	0	0	0	0	0	0	0	0	0	0	0	0	1	0	1	0	0	0	1	0	0	0	0	0
A2	Pandeiros	0	0	0	0	0	0	0	0	0	0	0	1	0	0	0	0	0	1	0	1	0	0	0	2	0	0	0	0	0
B1	Teles Pires	0	0	0	0	0	0	0	0	0	0	0	0	0	0	0	1	1	0	0	0	0	0	0	0	0	1	1	0	0
B2	Branco	1	2	0	2	2	0	0	0	0	0	2	0	0	0	0	1	1	0	0	0	0	0	0	0	0	1	1	0	0
B2	Água Fria	1	0	0	2	0	0	0	0	0	0	1	2	0	0	0	1	1	0	0	0	0	0	0	0	0	1	1	0	2
B2	Pitinga	1	0	0	3	0	0	0	0	0	0	1	2	0	0	0	1	1	0	0	0	0	2	0	0	0	1	1	0	0
B2	Caroebe	1	0	0	3	0	0	0	0	0	0	1	2	0	0	2	1	1	0	0	0	0	2	0	0	0	1	1	0	2
B2	Ereu	1	0	0	3	3	0	0	0	0	0	1	0	0	0	0	1	1	0	0	0	0	2	0	0	0	1	1	0	0
B2	Cachorro	1	0	0	3	0	0	0	0	0	0	1	2	0	0	2	1	1	0	0	0	0	0	0	0	0	1	1	0	2
B2	Maroni	1	0	0	2	0	0	0	0	0	0	1	2	0	0	0	1	1	0	0	0	0	0	0	0	0	2	1	0	2
B3	Tracajatuba	1	0	0	0	0	0	0	0	0	0	1	2	0	0	0	1	1	0	0	0	0	0	0	0	0	1	1	0	0
B3	Oiapoque	1	0	0	0	0	0	0	0	0	0	1	2	0	0	0	1	1	0	0	0	2	0	0	0	0	2	1	0	0
B4	Parupa	1	0	0	0	0	0	0	0	2	0	1	0	2	0	0	1	1	0	0	0	0	0	0	0	0	0	1	0	0
B4	Yuruani	1	2	0	0	0	0	0	0	2	0	1	0	2	0	0	1	1	0	0	0	2	0	0	0	0	0	1	0	0
D	Xingu	0	0	0	0	0	1	1	1	0	0	0	0	0	0	0	0	0	0	1	0	0	0	1	0	0	0	0	0	0
D	Iriri	0	0	0	0	0	1	1	1	0	0	0	0	0	0	0	0	0	0	1	0	0	0	2	0	0	0	0	0	0
E1	São José	0	0	0	0	0	0	0	0	0	0	0	0	0	1	0	0	0	0	0	0	0	0	0	0	0	0	0	0	0
E2	Araguaia	0	0	1	0	0	0	0	0	0	0	0	2	0	2	0	0	0	0	0	0	0	0	0	0	0	0	0	2	0
E3	Mortes	0	0	0	0	0	0	0	0	0	0	0	2	0	2	0	0	0	0	0	0	0	0	0	0	0	0	0	2	0
E3	Pardo	0	0	0	0	0	0	0	0	0	0	2	2	0	2	0	0	0	0	0	0	0	0	0	0	0	0	0	2	0
E4	Verdão	0	0	2	0	0	0	0	0	0	0	0	0	0	2	0	0	0	0	0	0	0	0	0	0	0	2	0	0	0
F	Jacareí	0	0	0	0	0	0	0	0	0	2	0	0	0	0	0	0	0	0	0	0	0	0	0	0	2	0	0	0	0
F	Pirangi	0	0	0	0	0	0	0	0	0	2	0	0	0	0	0	0	0	0	0	0	0	0	0	0	2	0	0	0	0
F	Longá	0	0	0	0	0	0	0	0	0	2	0	0	0	0	0	0	0	0	0	0	0	0	0	0	2	0	0	0	0

^1^ Character states for inversions: 0 = absent, 1 = fixed, 2 = autosomally polymorphic, and 3 = sex-linked. Character numbers correspond with inversions as follows: 1. IS-3, 2. IS-8, 3. IS-12, 4. IS-13, 5. IS-15, 6. IS-16, 7. IS-17, 8. IL-3, 9. IL-4, 10. IL-5, 11. IL-6, 12. IL-8, 13. IIS-1, 14. IIS-2, 15. IIS-3, 16. IIL-2, 17. IIL-3, 18. IIL-4, 19. IIL-5, 20. IIL-6, 21. IIL-10, 22. IIL-14, 23. IIL-18, 24. IIIL-3, 25. IIIL-6, 26. IIIL-7, 27. IIIL-9, 28. IIIL-11, 29. IIIL-12.

Our hypothesis of relationships showed that the two populations of cytoform D were supported as monophyletic by inversions *IS-16*, *IS-17*, *IL-3*, *IIL-5*, and IIL-18. The A group, composed of two clades (A1 and A2), was supported by inversions *IIL-4*, *IIL-6*, and IIIL-3. Cytoform F, which included three populations, was recovered as a monophyletic group, supported by two autosomal polymorphisms (IL-5 and IIIL-6). The E group, composed of four cytoforms, was supported as monophyletic by autosomal polymorphism IIS-2. Within this group, E1 and E4 were monophyletic clades, whereas E2 and E3 were not recovered as distinct lineages, but instead nested in a clade supported by inversions IL-8 and IIIL-11. IL-8 is hypothesized to have been an ancestral polymorphism at a deeper node that included members of the A, B, and E groups. As such, it persisted as a polymorphism in some lineages, was fixed in A2, and was lost in A1, B1, B2 (Branco and Ereu populations), B4, E1, and E4. The B group was recovered as monophyletic, supported by inversions *IIL-2*, *IIL-3*, *IIIL-9*, and IIIL-7, which was lost in B4. Within this group, each cytoform (B1, B2, B3, and B4) was recovered as monophyletic. B1 was sister to the other cytoforms of the B group. The clade composed of B2+B3+B4 was supported by fixed inversion *IS-3* and IL-6, which was fixed except in the Branco population of B2, in which it was an autosomal polymorphism. Two groups were recovered in B2: one formed by the Branco + Ereu populations, characterized by IS-15, and the other including the remaining five B2 populations, supported by autosomal polymorphism IL-8.

### Distributions of cytoforms

**Fig 17:**
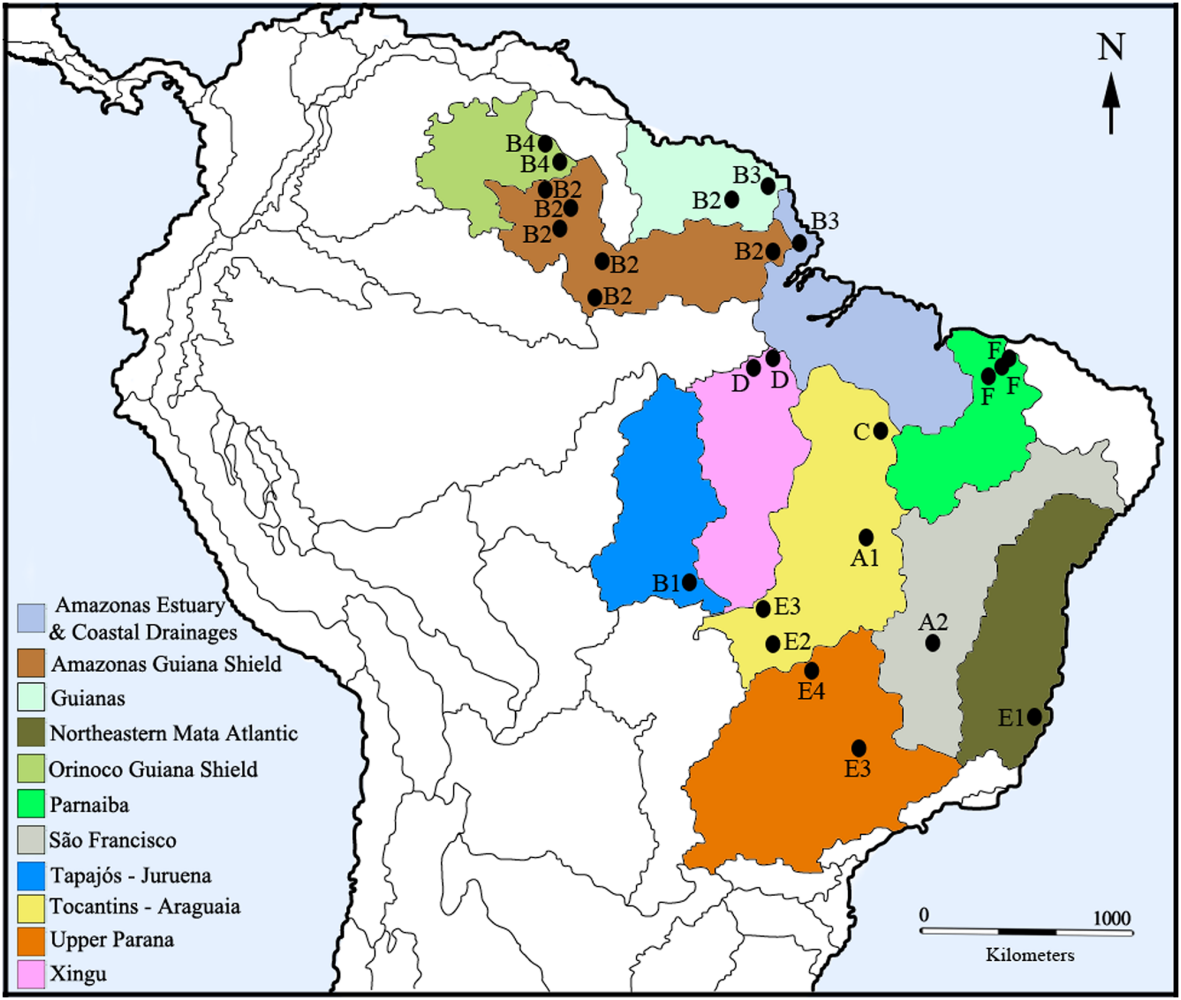
Freshwater ecoregions of South America [25] where cytoforms of *Simulium guianense s*. *l*. were collected.

Related cytoforms were clustered geographically, and each cytoform showed fidelity to a particular freshwater ecoregion (Fig 17). Only three of the 11 ecoregions represented by our collections harbored more than one cytoform: Guianas had two (B2 and B3), Upper Parana had two (E3 and E4), and Toncantins-Araguaia had four (A1, C, E2, and E3). The B group occupied the northwestern region of Brazil and adjacent areas related to the Guiana Shield: Orinoco Guiana Shield, Amazonas Guiana Shield, and Guianas. Only the populations of the Água Fria and Tracajatuba rivers (Amazonas Estuary & Coastal Drainages) and the geographically remote Teles Pires River (Tapajós-Juruena) were outside the Guiana Shield ecoregions. Although the B1 population was 300 km from the nearest other sampled population (cytoform E3 in the Mortes River), versus 1500 km from the nearest group B population (Pitinga River), it shared no inversions with cytoform E3.

## Discussion

### Interspecific relationships

*Simulium guianense s*. *l*. is currently placed in the *S*. *orbitale* group of the subgenus *Trichodagmia*, based on morphological criteria [15,37]. A molecular (COI) barcoding analysis of nine of the 18 nominal species in the *S*. *orbitale* group, including *S*. *guianense s*. *l*., however, did not recover this group [21]. We note, however, that the current concept [15] of the *S*. *orbitale* group includes taxa at one time contained in two separate subgenera, *Thyrsopelma* and *Grenieriella* [38].

Our unpublished chromosomal analysis of eight other members of the Brazilian *S*. *orbitale* group indicates that *S*. *guianense s*. *l*. can be derived from the group standard by at least 12 inversions and that at least eight of these inversions are uniquely shared by all cytoforms of the *S*. *guianense* complex. An earlier molecular (COI) analysis suggested that one member of the *S*. *orbitale* group, the morphologically similar *S*. *litobranchium* of Hamada et al. [20], clustered within *S*. *guianense s*. *l*. [22]. Our chromosomal analysis, however, unequivocally shows that *S*. *guianense s*. *l*. is monophyletic and that *S*. *litobranchium*, based on material from the type locality (Ponte de Pedra, Goiás State), is not a member of the *S*. *guianense* complex. Rather, it is removed from the standard banding sequence of the *S*. *guianense* complex by at least 10 fixed inversions. The chromosomal results find support in a morphological analysis showing *S*. *litobranchium* as the sister species of *S*. *perplexum* Shelley et al. and these two species as the sister group of *S*. *guianense s*. *l*. [37].

### Taxonomic status of cytoforms

Four of the 13 cytoforms (B1, D, E2, and E4) are each fixed for one or more unique inversions. The entire B group shares two unique fixed inversions. The most chromosomally differentiated cytoforms, B3 and D, are separated by nine fixed inversions, three nearly fixed inversions, and a geographic distance of less than 500 km. The geographically nearest cytoforms with at least one fixed-inversion difference are E2 (Araguaia River) and E3 (Mortes River), approximately 130 km apart, with no obvious topographical barriers to gene exchange. Although the chromosomal data suggest multiple species within *S*. *guianense s*. *l*., no opportunities were presented for using chromosomal features (e.g., fixed differences) in strict sympatry as the criterion for inferring reproductive isolation between any of the cytoforms. *Simulium metallicum s*. *l*., another Latin American vector associated with onchocerciasis, consists of 17 cytoforms [39–41]. About half of the cytoforms of *S*. *metallicum s*. *l*., however, have been found in sympatry with one another and confirmed as reproductively isolated species [39,41]. We suspect that, like *S*. *metallicum* and other Neotropical vector complexes such as *S*. *ochraceum* [42], *S*. *guianense* consists of multiple, reproductively isolated species. However, additional sampling of *S*. *guianense s*. *l*. is needed to minimize geographic gaps between cytoforms and allow rigorous tests of reproductive isolation.

The presence of shared inversions (IL-6, IL-8, and IIIL-7) between some members of the well-defined B and E groups suggests either gene exchange (introgression) or common ancestry. If the inversions reflect common ancestry, as we suggest, they exhibit the typical pattern in the Simuliidae: the ability of a single inversion to assume different states (e.g., fixation, loss, or polymorphism) in separate evolutionary lineages [29,39,43,44].

A previous molecular analysis of material from six of our sites, representing cytoforms B2, B3, C, E3, and E4, showed no pattern of clustering, with one exception [22]. The Farinha River population (cytoform C) clustered as a distinct group in a maximum likelihood tree based on the COI sequence. Of the five cytoforms molecularly evaluated [22], C is chromosomally the most remote. Thus, the molecular and chromosomal data are congruent for the most chromosomally divergent cytoform among those evaluated, but the chromosomal data provide finer resolution of the genetic structure of the other populations.

A few taxonomically critical collections are lacking. We do not have material from the type locality of *S*. *guianense* on the upper Essequibo River in Guyana to fix the name with the relevant cytoform. The type locality is within about 200 km of the Cachorro and Caroebe populations of cytoform B2, but it is in the Essequibo ecoregion, separate from all other ecoregions of our sampled populations. Although the type is probably a member of the B group, we hesitate to assign it to a particular cytoform, given the influence of ecoregion on simuliid distributions [45]. We also lack material from the type localities associated with the two names currently held in synonymy with *S*. *guianense*. The type specimen of *S*. *pintoi* from Piracicaba in São Paulo State is about 220 km from our nearest sampling site in the Pardo River, which supports cytoform E3. *Simulium ortizi* is based on material from San Felix in Bolívar State, Venezuela, roughly 315 km from our nearest sampling site in the Parupa River, which supports cytoform B4.

### Association of cytoforms with onchocerciasis

Only the B group has been found in Venezuela and the North Region of Brazil, the general area of the last-remaining Latin American onchocerciasis foci, suggesting that one (or more) of its members is the vector. Although we did not sample rivers within the foci, they are within a few hundred kilometers of the Ereu (cytoform B2) and Parupa (cytoform B4) populations, B2 having been collected only from the lowlands and B4 only from the uplands. A caveat is that elevation, in addition to river system, can influence gene flow in simuliids, even over elevational distances as little as several hundred meters [46]. Caution, therefore, is needed in assigning vector status to any known member(s) of the B group.

Shelley et al. [47] suggested that cytoform A1 was predominantly zoophilic and, therefore, not a vector in the Minaçu area of Brazil historically afflicted with onchocerciasis. We recorded *S*. *guianense s*. *l*. biting humans along the Tracajatuba River in the state of Amapá where onchocerciasis has never been a problem; the corresponding larval population was cytoform B3. The cytoform identity is unknown for populations of *S*. *guianense s*. *l*. in areas currently or historically associated with onchocerciasis. Relevant material, therefore, is critically needed from the New World’s last-remaining onchocerciasis foci along the border of Brazil and Venezuela. In other areas of Latin America, *S*. *metallicum s*. *l*. includes six cytoforms implicated as vectors through their geographic association with onchocerciasis foci, whereas the remaining cytoforms have been found only in onchocerciasis-free areas [39, 41]. The pattern is repeated in *S*. *ochraceum s*. *l*., another Latin American vector complex in which only a subset of cytoforms is associated with onchocerciasis [42].

### Spatial distribution patterns of cytoforms

The discrete distributions of the 13 cytoforms suggest an association with geographic factors that might govern the distributions. The Amazon River separates the B group, except B1, from all other cytoforms. A COI analysis of eight populations of *S*. *guianense s*. *l*. also indicated genetic differences between opposite sides of the Amazon River [19]. The role of the Amazon River as a barrier to dispersal is well documented for numerous taxa, including winged animals such as birds [48]. Our populations of *S*. *guianense s*. *l*. south of the Amazon River are chromosomally more divergent (i.e., more cytoforms) than those on the northern side, although the chromosomal diversity (i.e., number of different rearrangements) is nearly the same (45 south versus 49 north of the Amazon). The greater diversity of cytoforms (9 south versus 2 north) perhaps reflects greater habitat diversity, expressed as different ecoregions. Ecological diversity among closely related species of black flies has been suggested as an indication that ecological adaptation has been important in driving evolution in the Simuliidae [46,49].

Cytoforms in the Northeast Region of Brazil align more with areas of rainforest endemism [50] or with geography than with drainage basin. Cytoform F, for example, is represented by a geographic cluster of populations in an area of endemism in a small independent drainage (Parnaíba) flowing directly to the ocean. If drainage basin or ecoregion were universally important for *S*. *guianense s*. *l*., however, the Pardo and Verdão rivers should support similar chromosomal profiles, and the Araguaia, Farinha, Mortes, and Tocantins populations should group together. Based on drainage basin alone, the grouping of the Pardo and Mortes populations (cytoform E3) is puzzling. These populations, however, are weakly grouped based on an absence of diagnostic chromosomal rearrangements; additional (molecular?) study might reveal greater divergence.

The influence of physicochemical properties of rivers, such as pH and conductivity, on cytoform distributions is unknown, although these properties differ across the distribution of *S*. *guianense s*. *l*., with different cytoforms experiencing different physicochemical environments [51,52]. Environmental conditions also can influence some aspects of chromosomal expression. The degree of pairing of the chromosomal homologues, for example, is positively related to water temperature in at least some species of black flies in temperate regions [53]. Whether this scenario applies to *S*. *guianense s*. *l*. or is cytoform specific is not known. However, we observed the greatest degree of nonpairing in cytoform D, which was collected in the warmest waters (> 30°C)—a finding opposite our expectation, based on data [53] for temperate species.

The four cytoforms in the B group reflect a distributional series of nonoverlapping elevations: B3 at 7–14 m asl, B2 at 48–146 m, B1 at 356 m, and B4 at 870–1270 m. The cytoforms of *S*. *ochraceum s*. *l*., a vector complex involved with onchocerciasis in Central America, also exhibit distinct elevational distributions [42]. Typically, as elevation decreases, rivers become larger. River size is a strong predictor of distributions of many species [54], and cryptic species in a complex are often distributed along and among streams and rivers by size of the watercourse [55]. For *S*. *guianense s*. *l*., however, this association is weak, especially in the B group, which exhibits no correlation between river width and elevation (Spearman’s rho -0.070, P = 0.838). B2, for example, is found in the Caroebe River, about 20 m wide at 146 asl, and in the Branco River, roughly 600 m wide at 48 m asl.

Differential microdistribution patterns have been documented for larvae and pupae of isomorphic simuliid species [56]. However, different substrates used by the larvae of *S*. *guianense s*. *l*. in our study—leaves of Podostemaceae in the Jacareí River, rocks and leaves of Podostomaceae in the Pirangi River, and bedrock in the Longá River—cannot be attributed to cytoform; larvae in these three rivers were chromosomally homogeneous (cytoform F). Rather, we agree with Santos-Neto et al. [52] who suggested that the differences in substrate use are attributable to scarcity of the typical substrate, the leaves of Podostemaceae.

A striking pattern reflected in the chromosomal inversion profiles is the remarkable river specificity of the populations. Given the enormous populations [52] produced over multiple generations, potential dispersal capability, and apparent lack of topographical barriers to gene exchange, *S*. *guianense s*. *l*. might be expected to be genetically homogeneous across rivers over a wide geographic area. Yet, we find the opposite: populations of *S*. *guianense s*. *l*. are typically river specific. In one of the main onchocerciasis areas in southern Venezuela, for example, the immature stages of *S*. *guianense s*. *l*. inhabit a particular site (“Raudal of Goaharibos”) in the Orinoco River [57], even though adults of *S*.*guianense s*. *l*. are found in most Yanomami villages throughout the area [58]. What drives this specificity? Adler et al. [59] have argued that as watercourses become larger, they become less common and, therefore, less likely for females to find. Selection, therefore, should favor females that return to oviposit in natal habitats where larvae and pupae are adapted to the particular riverine conditions. The return would be especially advantageous to *S*. *guianense s*. *l*., which inhabits not only large rivers, but more specifically, the sporadically distributed rocky shoals of large rivers. Site fidelity would build site-specific genetic profiles, potentially leading to population differentiation, including speciation. As a corollary, within-river genetic diversity for *S*. *guianense s*. *l*. would be expected to be low [59]. The extreme examples of this prediction are found in cytoforms B1, C, and D, which express virtually no polymorphism.

Given the high degree of river specificity, environmental perturbations are likely to differentially influence the cytoforms of the *S*. *guianense* complex, potentially putting some cytoforms (species?) at risk. For a multivoltine complex of cytoforms, the consequences of natural fluctuations in water levels that might inundate or leave appropriate habitat dry raise questions about the responses of ovipositing females and other life stages over time. For example, does oviposition occur in the natal rivers during droughts and floods, and, if so, are the eggs able to survive these events? Studies of larval populations and their chromosomal inversion profiles within rivers over time could provide insights into the responses to these events and the degree of dispersal. The construction of dams on large rivers in Brazil also could have profound effects on populations of *S*. *guianense s*. *l*. as rocky shoals and riffle areas—prime breeding habitats—become permanently inundated. Substrate switching by the larvae of *S*. *guianense s*. *l*., for example, has been attributed to the construction of a dam in Amazonas [51]. Extinction is the extreme consequence of river specificity in the face of habitat alteration. Environmental disturbances, thus, carry greater risks for biodiversity than previously appreciated, particularly in light of the pattern that is coming to light in the Simuliidae: many so-called species represent taxonomic mosaics of forms and species ranging from pests and vectors to endangered species [59, 60].
